# Targeting NAT10 Inhibits Hepatocarcinogenesis via ac4C‐Mediated SMAD3 mRNA Stability

**DOI:** 10.1002/EXP.20250075

**Published:** 2025-09-04

**Authors:** Yigan Zhang, Yanbin Dong, Shuwen Chen, Hao Deng, Weiru Yu, Bonan Chen, Minjie Chen, Wanglong Liu, Xiao Tan, Jiaxin Ni, Daniel Rigden, Xuan Wang, Wuhua Zhou, Jia Meng, Juan Chen, Yuanchao Xue, Zhongji Meng

**Affiliations:** ^1^ Institute of Biomedical Research Department of Infectious Diseases Regulatory Mechanism and Targeted Therapy for Liver Cancer, Shiyan Key Laboratory Hubei Provincial Clinical Research Center for Precise Diagnosis and Treatment of Liver Cancer Taihe Hospital (First Clinical College of Medicine) Hubei University of Medicine Shiyan Hubei China; ^2^ Key Laboratory of RNA Biology, Institute of Biophysics, Chinese Academy of Sciences Beijing China; ^3^ Department of Pathology The Affiliated Lianyungang Hospital of Xuzhou Medical University The First People's Hospital of Lianyungang Lianyungang China; ^4^ Hubei Key Laboratory of Tumor Microenvironment and Immunotherapy College of Basic Medical Science China Three Gorges University Yichang P. R. China; ^5^ Key Laboratory of Precision Nutrition and Food Quality Department of Nutrition and Health China Agricultural University Beijing China; ^6^ Department of Anatomical and Cellular Pathology State Key Laboratory of Translational Oncology Sir Y. K. Pao Cancer Center Prince of Wales Hospital The Chinese University of Hong Kong Hong Kong China; ^7^ Department of Hepatobiliary Pancreatic Surgery Affiliated Taihe Hospital Hubei University of Medicine Shiyan City Hubei Province China; ^8^ School of Ophthalmology and Optometry and Eye Hospital Wenzhou Medical University Wenzhou China; ^9^ Department of Biochemistry Cell and Systems Biology Institute of Systems Molecular and Integrative Biology University of Liverpool Liverpool UK; ^10^ Department of Biological Sciences School of Science Xi’an Jiaotong‐Liverpool University Suzhou China

**Keywords:** liver cancer, RNA ac4C modification, targeted drug development

## Abstract

Hepatocellular carcinoma (HCC) is characterized by high morbidity and mortality, with limited effective treatment options. *N*‐acetyltransferase 10 (NAT10) is the only known acetyltransferase for mRNA ac4C modification and is recognized as a biomarker for HCC, promoting its progression. However, the critical role of NAT10 in hepatocarcinogenesis remains to be fully elucidated, and the identification of suitable small‐molecule inhibitors targeting NAT10 is of great interest. Here, we report that NAT10 promotes HCC progression by stabilizing SMAD family member 3 (SMAD3) mRNA through ac4C modification. Clinically, NAT10 is highly expressed in HCC tissues and is significantly associated with poor prognosis. Functionally, NAT10 downregulation inhibits HCC cell proliferation, invasion, and epithelial‐mesenchymal transition, while promoting anoikis in vitro. Additionally, NAT10 depletion significantly impairs tumor growth, metastasis, and hepatocarcinogenesis in vivo. Mechanistically, NAT10 enhances oncogene SMAD3 mRNA stability via ac4C modification, thereby activating TGF‐β signaling pathway. We also identify a novel small‐molecule inhibitor, NAT10‐2023, which effectively blocks NAT10 activity. Notably, NAT10‐2023 treatment significantly reduces intracellular RNA ac4C modification levels and disrupts NAT10‐RNA interactions, leading to suppressed tumor progression. Overall, NAT10 drives HCC progression via SMAD3 mRNA stability regulation, and NAT10‐2023 could be a promising therapeutic candidate for targeting NAT10 in cancer treatment.

## Graphical abstract

This study demonstrates that elevated *N*‐acetyltransferase 10 (NAT10) expression stabilizes SMAD3 mRNA through ac4C modification, promoting the progression of hepatocellular carcinoma (HCC). NAT10 also activates the TGF‐β signaling pathway, facilitating epithelial‐to‐mesenchymal transition and inhibiting anoikis. Furthermore, the authors develop a small‐molecule inhibitor, NAT10‐2023, which disrupts NAT10's interaction with RNA, reducing ac4C modification levels and suppressing HCC progression.

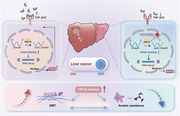

## 1 Introduction

RNA N4‐acetylcytidine (ac4C) modification is a conserved and widespread chemical alteration found across a diverse range of species, including thermophilic archaea, *Saccharomyces cerevisiae*, mice, and humans [1, 2, 3, 4, 5]. First identified by Arango et al. in 2018 using acRIP‐seq (N4‐acetylcytidine sequencing), ac4C has since become a focal point of research in RNA biology, particularly in the context of RNA metabolism and disease progression [6, 7]. Increasing evidence underscores the critical role of ac4C in RNA regulation, where it enhances mRNA stability and translation efficiency in a context‐dependent manner [6, 8, 9]. Specifically, ac4C promotes ribosome recruitment, induces structural rearrangements, and facilitates accurate decoding during protein synthesis. In disease contexts, the modification has been linked to several pathophysiological processes, including cancer progression, immune modulation, and chemoresistance [10, 11]. By stabilizing oncogenic transcripts and enhancing their translation, ac4C contributes actively to tumor growth, metastasis, and resistance to chemotherapy. The growing recognition of ac4C's biological significance thus positions it as a promising target for novel therapeutic strategies in cancer and other diseases.

*N*‐acetyltransferase 10 (NAT10) is currently the only known RNA acetyltransferase responsible for ac4C modification [12]. It is believed to regulate gene expression via the RNA ac4C modification pathway and thereby contribute to the development of various diseases [6, 8]. Additionally, NAT10 can participate in disease processes via non‐canonical RNA ac4C modification pathways [13]. As a crucial regulatory factor, NAT10 has been implicated in major human diseases including liver cancer, breast cancer, gastric cancer, colorectal cancer, and myocardial infarction [14, 15, 16, 17, 18, 19]. Given the growing recognition of RNA ac4C's role in tumor biology, several NAT10‐targeted drugs have been identified, such as Remodelin, paliperidone, and AG‐401 [20, 21]. Among these, Remodelin has been extensively studied and is considered a promising treatment option for diseases such as breast cancer and liver cancer [15, 22, 23, 24]. However, a study by Shrimp, J. H. et al. in 2020 questioned Remodelin's interaction with NAT10, asserting that it does not affect intracellular RNA ac4C modification levels [25]. Therefore, there is an urgent need to identify effective small‐molecule drugs targeting NAT10 and to explore their therapeutic potential in cancer treatment.

NAT10 and RNA N4‐acetylcytidine modification have attracted increasing interest in hepatocellular carcinoma (HCC) research. As early as 2015, several studies suggested that NAT10 could serve as a potential biomarker for HCC [26]. It has been shown to promote the metastasis of HCC cells and epithelial‐mesenchymal transition (EMT), thereby playing a crucial role in chemoresistance and cellular proliferation in HCC [22, 27]. Mechanistically, NAT10 exerts its effects through the ac4C modification pathway, which enhances the stability and translation of specific mRNAs, ultimately increasing their expression and accelerating progression of HCC both in vivo and in vitro [28, 29]. However, the development and progression of HCC is a complex and multifactorial process, influenced by a variety of external and internal factors. Despite significant progress, current research has yet to fully elucidate NAT10's regulatory role in HCC development, particularly in relation to hepatocarcinogenesis and oncogene activation.

Our study utilized various HCC models to demonstrate the essential role of NAT10 in promoting HCC development in vivo. Specifically, we revealed that NAT10 enabled HCC cells to proliferate, invade, migrate, and resist apoptosis through anoikis resistance and EMT regulation pathways. Mechanistically, NAT10 was found to directly interact with the 3′UTR of the key oncogene SMAD family member 3 (SMAD3) mRNA, introducing RNA ac4C modification to enhance its stability and upregulate its expression. In the context of drug discovery, we identified NAT10‐2023, a novel small‐molecule inhibitor of NAT10, which effectively inhibited NAT10 activity and suppressed HCC progression. Overall, our research not only elucidates the functional significance of NAT10 in HCC development but also introduces a potent NAT10 inhibitor, offering promising therapeutic potential for RNA ac4C modification‐related diseases, including HCC.

## 2 Results

### 2.1 NAT10 Upregulation Correlates With HCC Progression and Poor Prognosis

**FIGURE 1:**
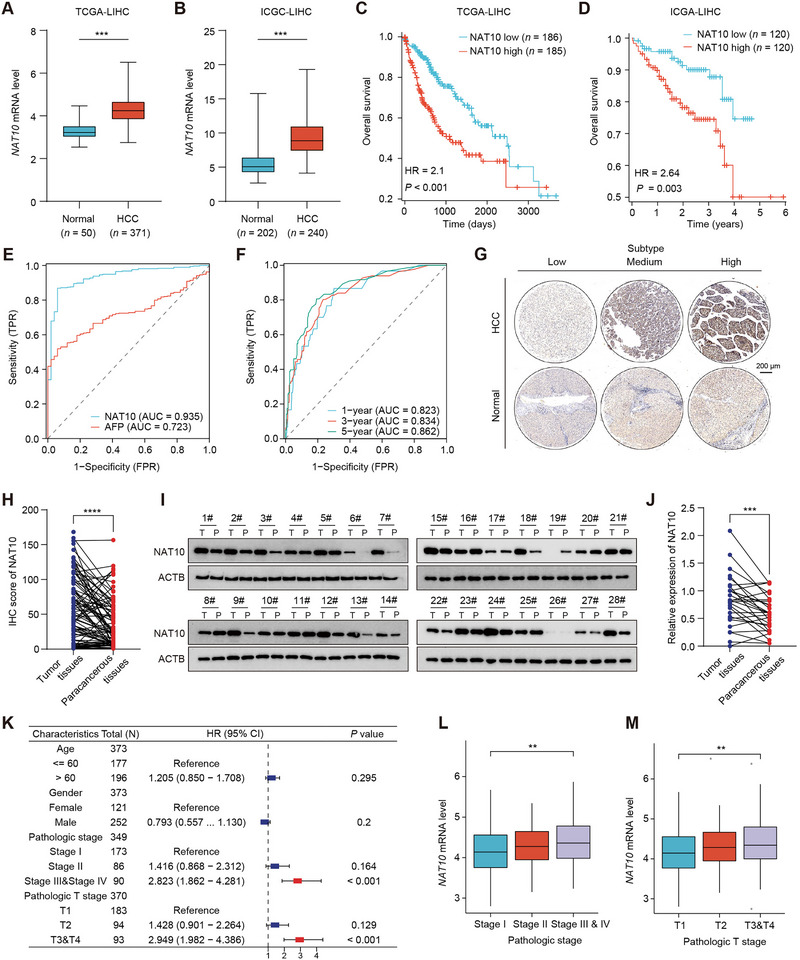
NAT10 is highly expressed in HCC and is associated with poor prognosis. (A,B) NAT10 mRNA expression in normal and HCC tissues from TCGA and ICGA databases. (C,D) Kaplan–Meier curves showing the relationship between NAT10 expression and overall survival in HCC patients from the TCGA and ICGA databases. (E) Diagnostic ROC curve of NAT10 and AFP in HCC patients from the TCGA database. (F) Time‐dependent ROC curves showing the ability of NAT10 expression to predict 1‐, 3‐, and 5‐year overall survival in HCC patients from the TCGA database. (G) Representative immunohistochemistry staining of NAT10 expression on HCC tissue microarrays. Scale bar, 200 µm. (H) Analysis of IHC score of NAT10 expression in tumor tissues (*n* = 95) and adjacent non‐tumor tissues (*n* = 87) from the HCC tissue microarrays. (I,J) WB analysis confirms the NAT10 expression in freshly paired tumor tissues (*n* = 28) and paracancerous tissues from HCC patients. (K) Univariate Cox regression analyses of risk factors affecting the survival of HCC patients. (L,M) The correlation between NAT10 mRNA expression level and pathological stage (L) and pathological T stage (M) in HCC patients. Data in (H) and (J) are presented as the mean ± SD. ^***^ *p* < 0.001, ^****^ *p* < 0.0001. Two‐tailed Student's *t*‐test. Abbreviations: HCC, hepatocellular carcinoma; IHC, immunohistochemistry; LIHC, liver hepatocellular carcinoma.

To explore NAT10's role in HCC, we first analyzed its expression and clinical significance using RNA‐seq data from HCC clinical samples from both The Cancer Genome Atlas (TCGA) and International Cancer Genome Consortium databases. The results demonstrated that NAT10 expression was significantly elevated in HCC tissues compared to normal counterparts in both databases (Figure 1A,B). Notably, Kaplan–Meier (K‐M) survival analysis further confirmed that patients with higher NAT10 expression exhibited significantly worse overall survival (OS) (Figure 1C,D). Moreover, receiver operating characteristic (ROC) curve analysis indicated that NAT10 expression could serve as a reliable diagnostic marker for HCC, with an area under the curve (AUC) value exceeding that of alfa‐fetoprotein (AFP) (AUC: 0.935 vs. 0.723) (Figure 1E). Furthermore, the time‐dependent ROC analysis supported the prognostic value of NAT10, with 1‐, 3‐, and 5‐year AUC values of 0.823, 0.834, and 0.862, respectively, demonstrating its ability to predict long‐term clinical outcomes in HCC patients (Figure 1F). Additionally, immunohistochemical (IHC) analysis of HCC tissue microarrays (TMA) revealed that NAT10 expression was significantly upregulated in 95 tumor tissues compared to 87 adjacent non‐tumor tissues from HCC specimens (Figure 1G,H). Similarly, in fresh tumor tissues from 28 HCC patients, Western blot (WB) analysis confirmed NAT10 upregulation in tumor tissues relative to paracancerous tissues (Figure 1I,J).

To further clarify the clinical implications of NAT10, we examined the relationship between its expression and the clinicopathological features of HCC. Specifically, clinical data from the TCGA database were subjected to univariate Cox regression analysis, revealing pathological stage and T stage as independent risk factors for patient survival (Figure 1K). Moreover, NAT10 mRNA expression was significantly associated with both the pathological stage and T stage, correlating positively with advanced disease progression in HCC patients (Figure 1L,M). Additionally, mRNA stemness index (mRNAsi) analysis revealed significant differences between patient groups classified based on NAT10 expression levels, with the high NAT10 group exhibiting higher mRNAsi scores compared to the low NAT10 group (Figure S1G). This finding suggests that elevated NAT10 expression is associated with enhanced stemness characteristics in HCC, indicating a more aggressive tumor phenotype. Furthermore, survival analysis demonstrated that HCC patients with high NAT10 expression exhibited significantly poorer disease‐specific survival and shorter progression‐free intervals (Figure S1H,I).

To gain deeper insights into the functional mechanisms of NAT10 in HCC, we performed gene ontology (GO) and Kyoto Encyclopedia of Genes and Genomes (KEGG) enrichment analyses on NAT10‐correlated genes from the TCGA database (Figure S1C–F). GO analysis revealed that genes positively correlated with NAT10 were enriched in biological processes such as RNA splicing, covalent chromatin modification, and histone modification, while negatively correlated genes were enriched in small molecule catabolic processes and acid catabolic processes (Figure S1C,D). KEGG analysis further demonstrated that positively correlated genes were involved in pathways related to the cell cycle, nucleocytoplasmic transport, and spliceosome (Figure S1E,F). Collectively, these findings suggest that NAT10 upregulation correlates closely with malignant HCC progression and poor prognosis.

### 2.2 NAT10 Facilitates Tumorigenic Potentials of HCC In Vitro and In Vivo

**FIGURE 2:**
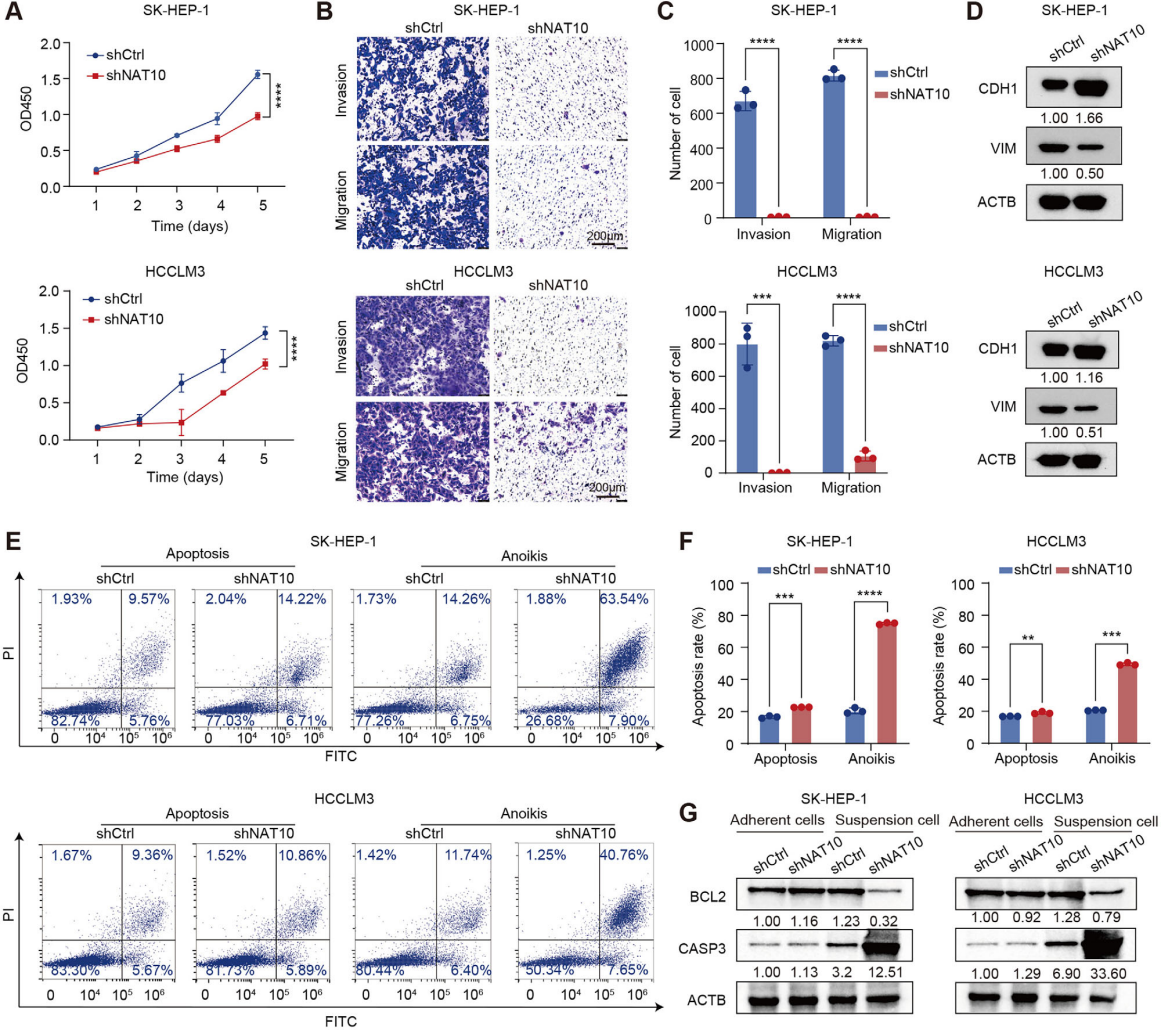
NAT10 knockdown inhibits HCC cell proliferation, invasion, and induces anoikis in vitro. (A) CCK8 assay showing the effect of NAT10 knockdown on cell proliferation in SK‐HEP‐1 and HCCLM3 cells. (B,C) Transwell assay shows the effect of NAT10 knockdown on invasion and migration in SK‐HEP‐1 and HCCLM3 cells. Scale bar, 200 µm. (D) WB showing the change of CDH1 and VIM protein levels upon NAT10 knockdown in SK‐HEP‐1 and HCCLM3 cells. (E,F) Flow cytometry shows the effect of NAT10 knockdown on cell apoptosis and anoikis in SK‐HEP‐1 and HCCLM3 cells. (G) WB validates the change of BCL2 and CASP3 expression under normal or low‐attachment condition upon NAT10 knockdown in SK‐HEP‐1 and HCCLM3 cells. Data in (A), (C) and (F) are presented as the mean ± SD (*n* = 3). ^**^ *p* < 0.01, ^***^ *p* < 0.001, ^****^ *p* < 0.0001. Two‐tailed Student's *t*‐test.

To investigate whether NAT10 exerted oncogenic roles in HCC, we first created HCC cell lines with NAT10 knockdown and assessed its effect on cancer cell phenotypes. Initial screening of NAT10 expression levels across seven commonly used HCC cell lines revealed significantly higher expression in SK‐HEP‐1 and HCCLM3 cells, as determined by WB and qPCR analyses (Figure S2A,B). Based on these findings, SK‐HEP‐1 and HCCLM3 cells were selected for further phenotypic validation. NAT10 expression was silenced using shRNA, and the knockdown efficiency was confirmed by WB and qPCR assays. The shRNA with the highest knockdown efficiency was used in subsequent experiments (Figure S2C–E). Functional assays demonstrated that NAT10 knockdown significantly inhibited HCC cell proliferation, as assessed by CCK8 assays (Figure 2A). Furthermore, the transwell assays confirmed that silencing NAT10 suppressed the invasion and migration abilities of HCC cells (Figure 2B,C). Moreover, we examined the changes in EMT marker expression upon NAT10 knockdown. WB results showed that silencing NAT10 upregulated E‐cadherin (CDH1) expression and downregulated vimentin (VIM) expression, suggesting that NAT10 influences the EMT process in HCC cells (Figure 2D).

We also examined the impact of NAT10 knockdown on apoptosis and observed that NAT10 knockdown significantly increased apoptosis rates in both SK‐HEP‐1 and HCCLM3 cells. Notably, under low‐attachment culture conditions, NAT10 knockdown significantly enhanced the apoptosis rates compared to standard culture conditions, indicating that NAT10 regulates anoikis in HCC cells (Figure 2E,F). Furthermore, we assessed the effects of NAT10 knockdown on key apoptotic markers. Consistent with the phenotypic results, under low‐attachment conditions, NAT10 knockdown resulted in a significant upregulation of the pro‐apoptotic marker CASP3 and a downregulation of the anti‐apoptotic marker BCL2 (Figure 2G). In contrast, no significant changes in these markers were observed under standard culture conditions. These findings suggest that NAT10 crucially influences anoikis resistance in HCC cells.

**FIGURE 3:**
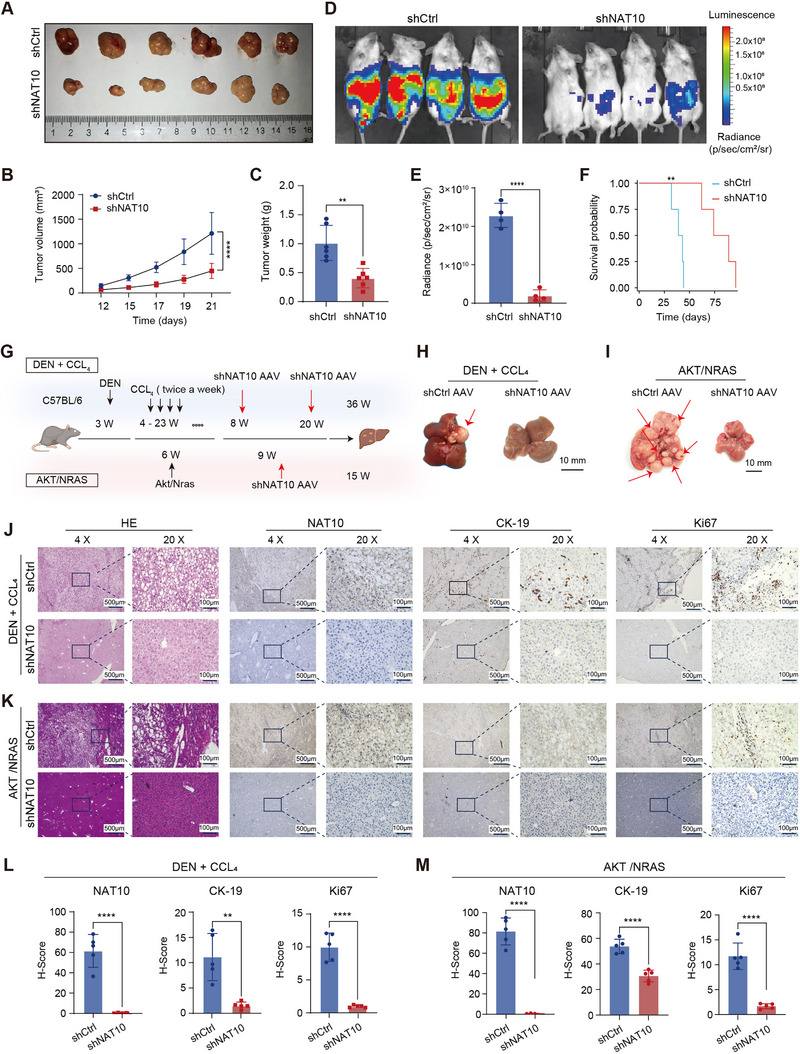
NAT10 knockdown attenuates tumorigenesis of HCC in vivo. (A) Representative images of tumors derived from mice with NAT10 knockdown and control groups of the HCC xenograft model (*n* = 6). (B,C) Tumor volume growth curves and tumor weight comparison between the NAT10 knockdown and control groups. (D) Bioluminescence images of mice in the intraperitoneal metastasis model of the NAT10 knockdown and control groups (*n* = 4). (E) Quantification of bioluminescence intensity in the NAT10 knockdown group compared to the control. (F) Effect of NAT10 knockdown on the overall survival of tumor‐bearing mice. (G) Schematic diagram of the DEN/CCL_4_ chemically induced HCC model and the AKT/NRAS oncogene‐driven HCC model used to assess hepatocarcinogenesis in C57BL/6 mice. (H,I) Representative images of liver tumors in the DEN/CCL_4_ and AKT/NRAS models showing the effect of NAT10 knockdown on tumor formation and growth. (J,K) Representative hematoxylin and eosin (HE) staining and immunohistochemistry (IHC) images for NAT10, CK‐19, and Ki67 in liver tissues from DEN/CCL_4_ (J) and AKT/NRAS (K) models following NAT10 knockdown. Scale bar, 100 µm. (L,M) Quantification of IHC results for NAT10, CK‐19, and Ki67 in DEN/CCL_4_ and AKT/NRAS and AKT/NRAS models following NAT10 knockdown. Data in (B),(C), (E), (L), and (M) are presented as the mean ± SD. ^*^ *p* < 0.05, ^**^ *p* < 0.01, ^***^ *p* < 0.001, ^****^ *p* < 0.0001. Two‐tailed Student's *t*‐test.

Subsequently, we assessed the impact of NAT10 on HCC proliferation and metastasis in vivo. First, tumor xenograft studies demonstrated that NAT10 silencing significantly inhibited tumor growth, with notable reductions in both tumor size and weight compared to the control group (Figure 3A–C). Furthermore, using an intraperitoneal metastasis model with HCCLM3‐Luciferase cells, we observed that NAT10 knockdown significantly suppressed metastasis and tumor growth in vivo (Figures 3D,E). Additionally, mice in the NAT10 knockdown group exhibited a substantial improvement in overall survival (Figure 3F), underscoring the critical role of NAT10 in promoting HCC progression and metastasis.

To further investigate the effect of NAT10 on hepatocarcinogenesis, we utilized two spontaneous HCC models in C57BL/6 mice: the DEN/CCL_4_ chemically induced model and the AKT/NRAS oncogene‐driven model (Figure 3G). In both models, NAT10 knockdown significantly impaired liver tumor formation, as evidenced by fewer and smaller tumors compared to the control mice (Figure 3H,I and Figure S3A,B). Histological analysis via HE staining revealed reduced tumor burden, while IHC analysis demonstrated significantly lower expression levels of tumor biomarkers CK‐19 and Ki67 in liver samples from the NAT10 knockdown group (Figure 3J–M). Additionally, WB results confirmed that NAT10 knockdown markedly reduced CK‐19 expression (Figure S3C–G). These findings collectively indicate that NAT10 plays a crucial role in promoting HCC initiation and progression. Moreover, NAT10 knockdown significantly improved liver function in both models, as evidenced by reduced serum ALT and AST levels (Figure S3H). Sirius Red staining in the DEN/CCL_4_‐induced model showed that NAT10 knockdown effectively suppressed liver fibrosis progression (Figure S3I,J). This antifibrotic effect was accompanied by significant reductions in serum AFP levels and pro‐inflammatory cytokines, including IL‐1β and IL‐6, as revealed by ELISA analysis (Figure S3K,L). These findings suggest that NAT10 inhibition not only reduces tumor biomarkers but also alleviates fibrosis by attenuating chronic inflammation. Collectively, these findings indicate that NAT10 plays the pivotal role as an oncogenic driver in HCC proliferation, metastasis, and hepatocarcinogenesis, both in vitro and in vivo.

### 2.3 NAT10 Regulates Oncogene SMAD3 mRNA Stability

**FIGURE 4:**
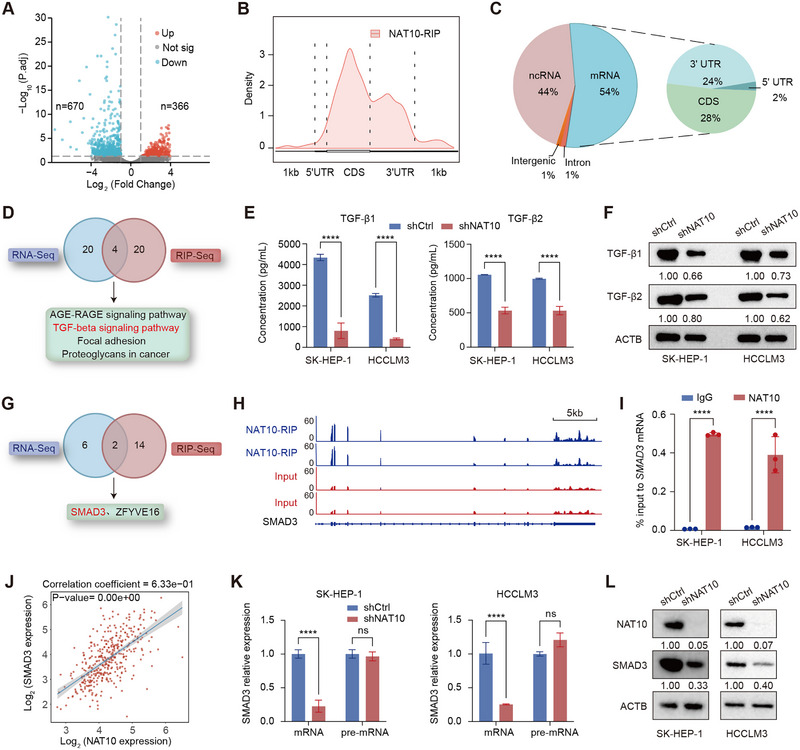
NAT10 regulates oncogene SMAD3 mRNA stability. (A) Volcano plot showing differentially expressed genes (DEGs) upon NAT10 knockdown in SK‐HEP‐1 cells. (B) Pie charts illustrating the proportion of NAT10 binding sites across the genome and mRNA. (C) Density plots showing the distribution of NAT10 binding sites across different mRNA regions. (D) Venn diagram integrating NAT10 knockdown RNA‐seq and NAT10 RIP‐seq identifies key enriched pathways. (E,F) ELISA and WB analyses demonstrating TGF‐β1 and TGF‐β2 protein levels upon NAT10 knockdown in HCCLM3 and SK‐HEP‐1 cells. (G) Venn diagram showing overlapped genes between NAT10 knockdown RNA‐seq and NAT10 RIP‐seq upon TGF‐β signaling pathway. (H) NAT10 RIP‐seq tracks showing NAT10 binding at the 3′UTR of SMAD3 mRNA. (I) RIP‐qPCR showing NAT10‐SMAD3 mRNA interaction in HCCLM3 and SK‐HEP‐1 cells. (J) Correlation plot showing positive correlation between NAT10 and SMAD3 mRNA expression. (K) qPCR analysis of SMAD3 pre‐mRNA and mRNA levels in SK‐HEP‐1 and HCCLM3 cells following NAT10 knockdown. (L) WB analysis of SMAD3 protein levels in SK‐HEP‐1 and HCCLM3 cells following NAT10 knockdown. Data in (I) and (K) are presented as the mean ± SD (*n* = 3). ^****^ *p* < 0.0001; ns, not significant. Two‐tailed Student's *t*‐test.

To explore the mechanism underlying NAT10's involvement in HCC tumorigenicity, we first noted that NAT10 enhances mRNA stability through its interaction with heterogeneous nuclear ribonucleoprotein Q (HNRNPQ) [30]. To further explore this, we performed RNA sequencing (RNA‐seq) following NAT10 knockdown in SK‐HEP‐1 cells. NAT10 depletion significantly altered gene transcription levels, leading to the identification of 366 upregulated and 670 downregulated NAT10‐related differentially expressed genes (DEGs), with a threshold of |log_2_FC| > 1 and adjusted *p* < 0.05 (Figure 4A). A heatmap was generated to illustrate the top 20 upregulated and downregulated DEGs (Figure S4A). Given that NAT10 knockdown predominantly led to downregulated gene expression, we propose that NAT10 plays a key role in regulating HCC cell progression through mRNA stability. Next, KEGG pathway enrichment analysis of the downregulated genes revealed significant enrichment in TGF‐β signaling and ECM‐receptor interaction pathways (Figure S4B). These pathways, which are closely associated with cancer progression, strongly suggest that NAT10 promotes HCC tumorigenicity by regulating these oncogenic signaling networks.

To identify direct RNA targets of NAT10 in HCC cells, we performed NAT10 RIP‐seq in SK‐HEP‐1 cells. The immunoprecipitation (IP) efficiency was validated by significant enrichment of NAT10 protein using a NAT10‐specific antibody compared to the IgG control (Figure S5A). The biological replicates of the NAT10 RIP‐seq library exhibited high reproducibility, with a Pearson correlation coefficient of 0.99 (Figure S5B). Subsequent genome‐wide analysis of NAT10 binding sites revealed that these sites were predominantly distributed in mRNA (52%) and ncRNA (44%). Within mRNA, NAT10 binding was primarily located in the coding sequence (CDS, 28%) and 3′UTR (25%) regions (Figure 4B,C). Additionally, motif analysis identified significant enrichment of the “CXXC” consensus motif in NAT10 binding sequences (Figure S4C), consistent with previous findings [17]. For further functional investigation, we identified 1843 high‐confidence target mRNAs for KEGG pathway enrichment analysis. These targets were significantly enriched in critical pathways, including mTOR, and TGF‐β signaling (Figure S4D). Importantly, integration of the top 20 enriched pathways from RNA‐seq and RIP‐seq analyses revealed four overlapping pathways: AGE‐RAGE signaling in diabetic complications, TGF‐β signaling, focal adhesion, and proteoglycans in cancer (Figure 4D). Given the pivotal role of TGF‐β signaling in cancer progression, fibrosis, and anoikis [31, 32], we selected this pathway for subsequent investigations.

We further investigated NAT10's role in regulating the TGF‐β signaling pathway by assessing its impact on the expression of TGF‐β1 and TGF‐β2. ELISA and WB analyses revealed that NAT10 knockdown significantly reduced TGF‐β1 and TGF‐β2 protein levels (Figure 4E,F), suggesting that NAT10 modulates HCC progression via TGF‐β signaling. To identify downstream targets, we intersected genes within the TGF‐β signaling pathway that were both downregulated in RNA‐seq and identified as targets in RIP‐seq. This analysis revealed two candidates: Zinc‐finger FYVE domain‐containing protein 16 (ZFYVE16) and SMAD3 (Figure 4G). Notably, literature evidence underscores the critical role of SMAD3 in liver cancer progression, prompting us to focus on SMAD3 as the key downstream target of NAT10 in subsequent investigations. Further analysis revealed that NAT10 binds to SMAD3 mRNA across the coding region, with additional enrichment at the 3′UTR (Figure 4H). Using NAT10 RIP‐qPCR, we validated the interaction between NAT10 and SMAD3 mRNA in both HCCLM3 and SK‐HEP‐1 cells (Figure 4I). Correlation analysis of the TCGA liver cancer database revealed a positive association between the expression levels of NAT10 and SMAD3 (Figure 4J). Interestingly, while NAT10 knockdown did not affect SMAD3 pre‐mRNA levels, it significantly reduced SMAD3 mRNA and protein levels (Figure 4K,L). These findings suggest that NAT10 plays a pivotal role in stabilizing SMAD3 mRNA, thereby enhancing its expression and promoting HCC progression.

### 2.4 NAT10 Regulates SMAD3 mRNA Stability via ac4C Modification

**FIGURE 5:**
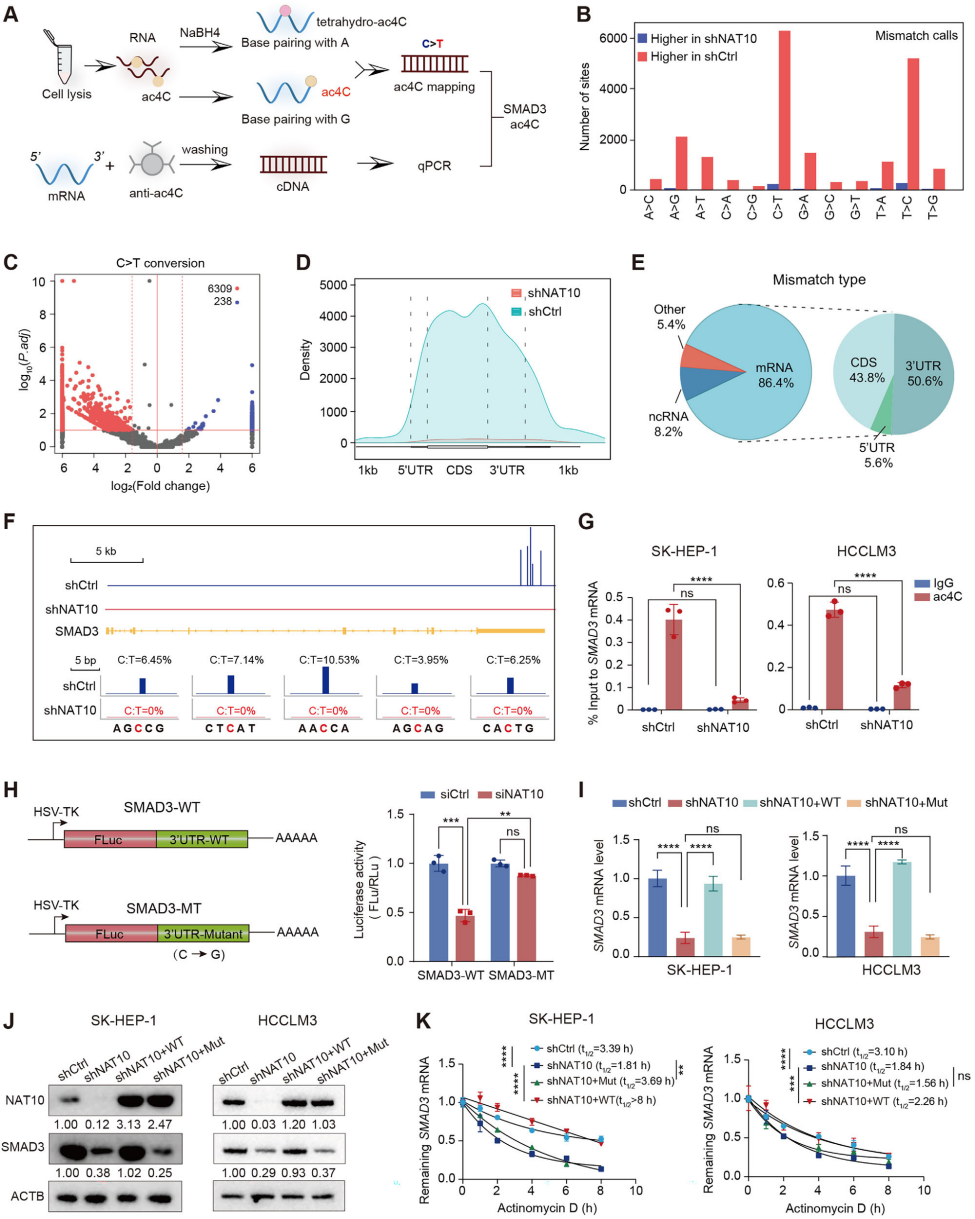
NAT10 regulates oncogene SMAD3 mRNA stability via ac4C modification. (A) Schematic illustration of Reda C:T‐seq technology used to detect ac4C modifications at single‐nucleotide resolution in NAT10‐depleted SK‐HEP‐1 cells. (B) mismatch frequencies showing a reduction in cytidine‐to‐thymidine (C > T) conversions in NAT10‐depleted cells. (C) Volcano plot showing differentially C > T mismatch between NAT10‐depleted and control cells. (D) Density plots showing the distribution of ac4C sites across different mRNA regions in control and NAT10‐depleted cells. (E) Pie charts illustrating the proportion of ac4C modifications in mRNA and their distribution in CDS and 3′UTR regions. (F) Schematic representation of SMAD3 C > T mismatch sites in control and NAT10‐depleted cells. (G) acRIP‐qPCR validation of ac4C modifications on SMAD3 mRNA in NAT10‐depleted SK‐HEP‐1 and HCCLM3 cells. (H) The left panel shows a schematic representation of luciferase reporter constructs with the SMAD3 3′UTR, including wild‐type (SMAD3‐WT) and mutant (SMAD3‐MT) versions where all five ac4C sites were mutated (cytidine‐to‐guanine); The right panel illustrates the effect of NAT10 knockdown on luciferase activity in the SMAD3‐WT and SMAD3‐MT constructs. (I) qPCR analysis showing SMAD3 expression after NAT10 knockdown and reintroduction of NAT10‐Wt or NAT10‐Mut in SK‐HEP‐1 and HCCLM3 cells. (J) WB analysis confirming changes in SMAD3 protein levels in response to NAT10 knockdown and reintroduction of NAT10‐WT or NAT10‐Mut in SK‐HEP‐1 and HCCLM3 cells. (K) RNA stability assays demonstrating changes in SMAD3 mRNA degradation after NAT10 knockdown and reintroduction of NAT10‐WT or NAT10‐Mut in SK‐HEP‐1 and HCCLM3 cells. Data in (G–I) and (K) are presented as the mean ± SD (*n* = 3). ^**^ *p* < 0.01, ^***^ *p* < 0.001, ^****^ *p* < 0.0001; ns., not significant. Two‐tailed Student's *t*‐test and one‐way ANOVA with Tukey's test.

Given the established role of NAT10 in modulating RNA stability through ac4C modification, we employed Reda C:T‐seq technology to explore transcriptome‐wide changes in RNA ac4C modifications at single‐nucleotide resolution in NAT10‐depleted SK‐HEP‐1 cells (Figure 5A). To characterize these changes, we analyzed mismatch frequencies from Reda C:T‐seq, focusing specifically on cytidine‐to‐thymidine (C > T) conversions, which are characteristic of ac4C modification mapping. As expected, C > T conversions were the predominant mismatch type (Figure 5B). Moreover, the biological replicates for both WT and NAT10 knockdown Reda C:T‐seq libraries exhibited extremely high correlations, with Pearson correlation coefficients exceeding 0.99 (Figure S5C). Notably, a substantial reduction in C > T mismatch frequency was observed in NAT10‐deficient SK‐HEP‐1 cells compared to controls, with 6309 sites exhibiting downregulated ac4C modification and only 238 sites showing upregulation (Figure 5C). Analysis of the distribution of ac4C modification sites revealed that these modifications were predominantly located in mRNA, accounting for 86.4% of all identified sites (Figure 5D,E). Within mRNA, ac4C sites were mainly enriched in CDS (43.59%) and the 3′UTR (50.6%) regions (Figure 5E). Importantly, a sequence motif enriched in cytidines (C‐rich) was disrupted in the NAT10 knockdown group, indicating a loss of ac4C recognition (Figure S5D). These findings highlight the critical role of NAT10 in mediating RNA ac4C modifications and its impact on transcriptome‐wide stability and functionality.

We then focused on SMAD3, a key downstream target of NAT10. Reda C:T‐seq analysis identified five distinct ac4C modification sites within the 3′UTR of SMAD3 mRNA in wild‐type SK‐HEP‐1 cells. However, these ac4C signals were completely lost following NAT10 knockdown (Figure 5F). To assess the abundance of ac4C modifications on SMAD3 mRNA, we determined that approximately 30.1% of SMAD3 transcripts harbored ac4C modifications (Figure S5E). To validate these observations, we performed acRIP‐qPCR in both SK‐HEP‐1 and HCCLM3 cells. The results confirmed a significant reduction in ac4C modification on SMAD3 mRNA following NAT10 depletion, consistent with the Reda C:T‐seq data (Figure 5G). To elucidate whether NAT10 directly regulates SMAD3 mRNA stability through ac4C modification, we conducted luciferase reporter assays targeting the ac4C sites within the 3′UTR of SMAD3 mRNA. Specifically, all five ac4C sites (cytidine‐to‐guanine mutations) were mutated to evaluate their functional significance. Interestingly, NAT10 knockdown significantly reduced luciferase activity in reporters containing the wild‐type ac4C peak region, while no such reduction was observed in reporters with mutated cytidine residues, suggesting that the ac4C modifications in the 3′UTR are essential for maintaining SMAD3 mRNA stability (Figure 5H). Additionally, we generated a NAT10‐G641E mutant (NAT10‐Mut), which lacks ac4C modification activity, as previously reported [33]. Both qPCR and WB analyses revealed that NAT10 knockdown significantly reduced SMAD3 mRNA and protein levels in SK‐HEP‐1 and HCCLM3 cells. Reintroduction of the shRNA‐resistant synonymous mutant of NAT10 (NAT10‐WT) successfully restored SMAD3 expression, whereas the NAT10‐Mut failed to rescue SMAD3 expression, underscoring the critical role of ac4C modification in regulating SMAD3 mRNA stability (Figure 5I,J). Finally, RNA stability assays demonstrated that SMAD3 mRNA degradation was markedly accelerated in NAT10‐depleted cells. Reintroduction of NAT10‐wt reversed the enhanced degradation, restoring SMAD3 mRNA stability, while NAT10‐mut was unable to rescue this effect (Figure 5K). These results conclusively demonstrate that NAT10 promotes SMAD3 mRNA stability through ac4C modification, playing a pivotal role in HCC progression by enhancing SMAD3 mRNA.

### 2.5 NAT10‐SMAD3 Regulatory Axis Enhance HCC Progression In Vitro and In Vivo

**FIGURE 6:**
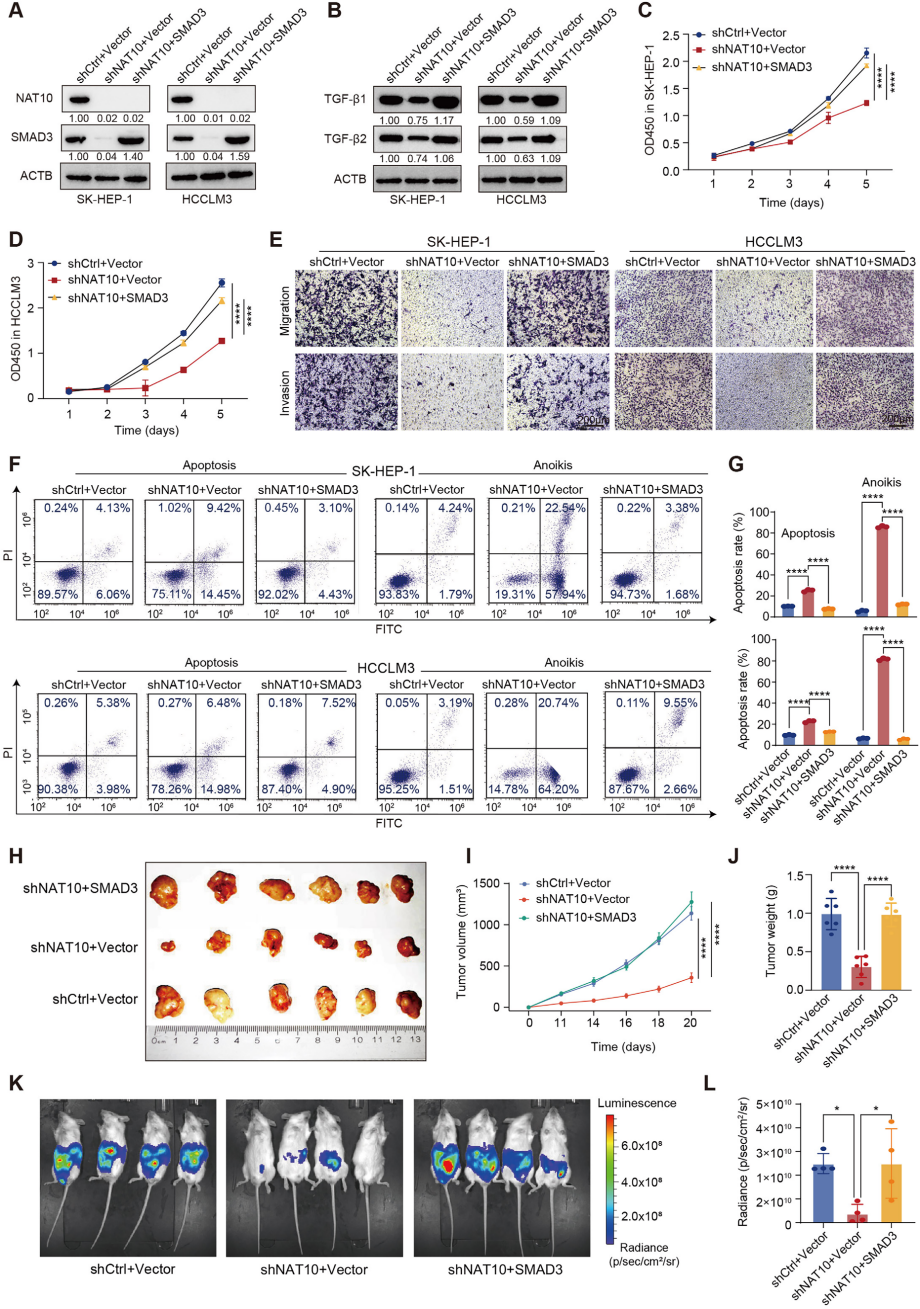
NAT10‐SMAD3 regulatory axis enhances HCC progression in vitro and in vivo. (A) WB analysis of NAT10 and SMAD3 protein levels after NAT10 knockdown and SMAD3 rescue in SK‐HEP‐1 and HCCLM3 cells. (B) WB analysis showing the expression levels of TGF‐β1 and TGF‐β2 after NAT10 knockdown and SMAD3 rescue in SK‐HEP‐1 and HCCLM3 cells. (C,D) Cell proliferation assays demonstrating the restoration of proliferation rates in SMAD3‐rescue groups of SK‐HEP‐1 and HCCLM3 cells. (E) Representative images of migration and invasion assays in SK‐HEP‐1 and HCCLM3 cells with NAT10 knockdown and SMAD3 rescue. (F,G) Flow cytometry analysis of apoptosis and anoikis in SK‐HEP‐1 and HCCLM3 cells following NAT10 knockdown and SMAD3 rescue. (H) Representative images of tumors from mouse xenograft models showing the effect of NAT10 knockdown and SMAD3 rescue (*n* = 6). (I,J) Tumor volume and weight measurements from mouse xenograft models showing the effect of NAT10 knockdown and SMAD3 rescue. (K) Bioluminescence images of mice in the intraperitoneal metastasis model of the NAT10 knockdown and SMAD3 rescue (*n* = 4). (L) Quantification of bioluminescence intensity in the NAT10 knockdown group compared to the SMAD3 rescue. Data in (C), (D), (G), (I), (J), and (L) are presented as the mean ± SD. ^*^ *p* < 0.05; ^****^ *p* < 0.0001; ns, not significant. One‐way ANOVA with Tukey's test.

To further elucidate the role of NAT10 in HCC progression and assess whether SMAD3 functions as a downstream mediator, we performed SMAD3 rescue assays in SK‐HEP‐1 and HCCLM3 cells following NAT10 knockdown (Figure 6A). Overexpression of SMAD3 significantly restored the expression of key molecules in the TGF‐β signaling pathway, including TGF‐β1 and TGF‐β2, which were reduced upon NAT10 depletion (Figure 6B and Figure S6A). Additionally, SMAD3 rescue assays showed that reintroducing SMAD3 could partially reverse the effects of NAT10 knockdown on several critical processes involved in HCC progression, including cell proliferation, invasion, EMT disruption, apoptosis, and anoikis in vitro. In terms of proliferation, SMAD3 overexpression in NAT10‐deficient cells restored reduced proliferation rates, as evidenced by cell viability assays (Figure 6C,D). Moreover, the transwell assays demonstrated that the impaired migratory and invasive capacities of NAT10 knockdown cells were significantly improved following SMAD3 overexpression (Figure 6E and Figure S6B). WB analysis confirmed that SMAD3 overexpression reduced CDH1 expression while restoring VIM expression, reversing the effects of NAT10 knockdown on EMT markers (Figure S6C). Flow cytometry and WB assays also demonstrated that SMAD3 reintroduction reduced apoptosis and anoikis rates in NAT10‐deficient cells (Figure 6F,G and Figure S6D).

To substantiate these in vitro findings, we conducted in vivo xenograft experiments. SMAD3 overexpression fully rescued the tumorigenic capacity of NAT10‐knockdown cells in the mouse xenograft model, with a marked increase in both tumor volume and weight in the SMAD3‐rescue group (Figures 6H–J). Furthermore, bioluminescence imaging and analysis in the intraperitoneal metastasis model confirmed that SMAD3 overexpression rescued the majority of the reduced tumor metastasis and growth caused by NAT10 knockdown (Figure 6K,L). These observations highlight SMAD3 as a critical downstream effector of NAT10 and underscore the significance of the NAT10‐SMAD3 regulatory axis in promoting HCC progression. Targeting the NAT10‐SMAD3 axis may present a promising therapeutic strategy for HCC.

### 2.6 Virtual Screening and Molecular Validation Reveal NAT10‐2023 as a Lead Compound for NAT10 Inhibition

**FIGURE 7:**
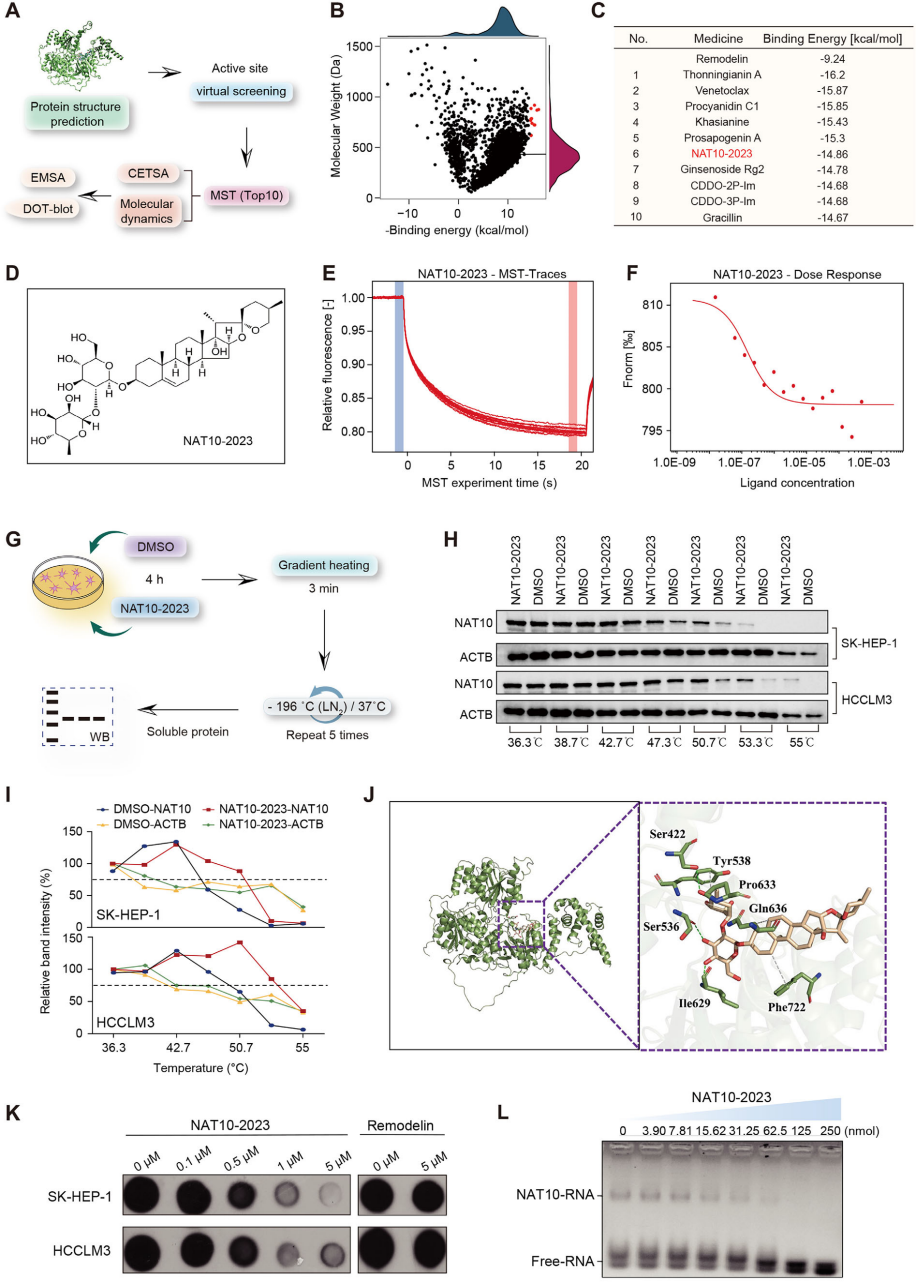
Identification and validation of NAT10‐2023 as a novel NAT10 inhibitor. (A) Schematic workflow of virtual screening and experimental validation to identify NAT10 inhibitors. (B) Scatter plot showing molecular weight and binding energy distribution of screened compounds. The red markings indicate the top 10 candidates based on binding energy. (C) Top 10 compounds ranked by binding energy compared to Remodelin. (D) Chemical structure of NAT10‐2023. (E) MST traces for NAT10‐2023, indicate a strong interaction with NAT10. (F) Dose‐response curve for NAT10‐2023 binding to NAT10. (G) Schematic representation of the cell thermal shift assay (CETSA) workflow. (H) WB showing the effects of NAT10‐2023 on NAT10 protein stability across different temperatures in SK‐HEP‐1 and HCCLM3 cells. (I) Quantification of soluble NAT10 protein levels from CETSA experiments. (J) Binding mode analysis of NAT10‐2023, highlighting key interacting residues. (K) Dot‐blot assay demonstrating NAT10‐2023's inhibition of RNA ac4C modification. (L) EMSA showing disruption of NAT10‐RNA interactions by NAT10‐2023.

Given the limitations of previously reported NAT10 inhibitors, such as Remodelin [25], whose interaction with NAT10 and inhibitory effect on RNA ac4C modification have been debated, we sought to identify a novel and potent NAT10 inhibitor through virtual screening approaches. Using AlphaFold2 to predict the NAT10 protein structure with high confidence, followed by molecular docking simulations via the AutoDock algorithm, we screened a library of over 4300 anti‐cancer small molecules from MCE to calculate their binding affinities with NAT10 (Figure 7A,B). Among the top 10 compounds ranked by binding energy, Remodelin exhibited a comparatively higher binding energy, suggesting that the newly identified small molecules may serve as superior NAT10 inhibitors (Figure 7C).

To experimentally validate the interaction between NAT10 and the top candidate compounds, we expressed recombinant NAT10 protein in HEK293 cells and performed microscale thermophoresis (MST) assays. Among the tested compounds, a newly identified molecule, NAT10‐2023, demonstrated a strong interaction with NAT10, with a dissociation constant (Kd) of 0.12 µM and a signal‐to‐noise ratio of 7.4. In contrast, Remodelin did not exhibit significant binding (Figure S7A–C and Figure 7D–F). Further investigation using cell thermal shift assays showed that NAT10‐2023 markedly increased the levels of soluble NAT10 protein in SK‐HEP‐1 and HCCLM3 cells at 47.3°C, 50.7°C, 53.3°C, and 55°C, compared to DMSO controls, indicating a robust and specific interaction with NAT10. Importantly, beta‐actin (ACTB) levels remained stable across all groups, confirming that the observed effects were specific to NAT10 stabilization (Figure 7G–I).

Moreover, molecular dynamics simulations revealed that the NAT10‐2023 and NAT10 complex achieved stability with root mean square deviation fluctuations around 2 Å after 28 ns, indicating the formation of a stable complex (Figure S7D). Hydrogen bond analysis of the molecular dynamic trajectory demonstrated that the compound formed an average of three hydrogen bonds with the protein, maintaining this interaction consistently throughout the simulation (Figure S7E). Key residues involved in these interactions were identified as Ser536, Ser422, and Ile629, with interaction frequencies of 83.56%, 49.19%, and 28.39%, respectively, highlighting their critical role in stabilizing the NAT10‐2023 interaction (Table S1). Binding mode analysis of the final simulation frame revealed that NAT10‐2023 fits snugly into a cleft in the protein through hydrophobic and hydrogen bonding interactions. Specifically, the glucopyranoside and hydroxylated six‐membered ring of NAT10‐2023 insert into the cleft, forming hydrogen bonds with Ser422, Ser536, and Ile629 at a distance of 2.7–2.9 Å. Additionally, the spirostan structure extends outside the cleft, facilitating hydrophobic interactions with residues Tyr538, Pro633, Gln636, and Phe722 (Figure 7J and Figure S7F).

Functional validation experiments confirmed that NAT10‐2023 significantly inhibited RNA ac4C modification at 1 µM concentration, as demonstrated by dot blot assays, whereas Remodelin did not exhibit a similar effect even at 10 µM (Figure 7K). Furthermore, electrophoretic mobility shift assays (EMSA) showed that NAT10‐2023 effectively disrupted NAT10‐RNA interactions at a concentration as low as 62.5 nM, highlighting its efficacy as a potent NAT10 inhibitor (Figure 7L). Collectively, these findings establish NAT10‐2023 as a novel small‐molecule inhibitor with high affinity and functional inhibition of NAT10, presenting significant potential for therapeutic applications targeting NAT10‐mediated pathways.

### 2.7 NAT10‐2023 Effectively Inhibits Tumorigenic Potentials of HCC In Vitro and In Vivo

**FIGURE 8:**
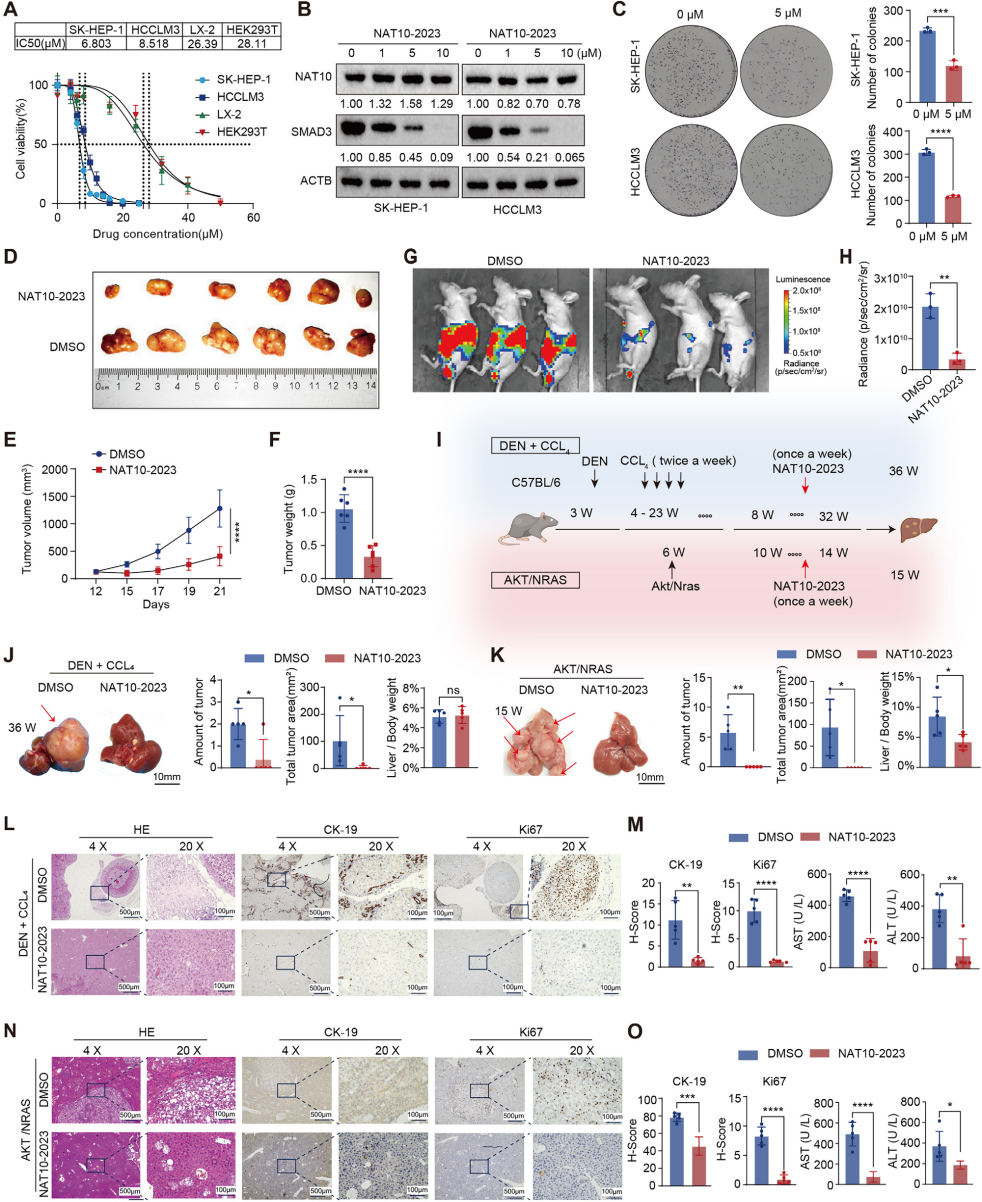
Identification in vitro and in vivo antitumor efficacy of NAT10‐2023. (A) Dose‐response curves showing cell viability in different cell lines treated with NAT10‐2023, with corresponding IC50 values. (B) WB analysis of NAT10 and SMAD3 expression in SK‐HEP‐1 and HCCLM3 cells treated with increasing concentrations of NAT10‐2023. (C) Colony formation assays demonstrating the effect of NAT10‐2023 on cell proliferation in HCCLM3 and SK‐HEP‐1 cells at 5 µM. (D) Representative images of tumors from xenograft models treated with NAT10‐2023 or DMSO (*n* = 6). (E,F) Tumor volume growth curves and tumor weight comparison between the NAT10‐2023 and DMSO groups. (G) Bioluminescence images of mice in the intraperitoneal metastasis model of the NAT10‐2023 and DMSO groups (*n* = 3). (H) Quantification of bioluminescence intensity in the NAT10‐2023 group compared to the control. (I) Schematic of the DEN/CCL_4_ and AKT/NRAS‐induced liver cancer models and NAT10‐2023 treatment timeline. (J,K) Representative liver images from DEN/CCL_4_ and AKT/NRAS models showing tumor reduction with NAT10‐2023 treatment. Quantitative analysis of the amount of tumor (left), the total tumor area (middle), and the ratio of liver weight to body weight (right) in the DEN/CCL_4_ and AKT/NRAS models upon NAT10‐2023 treatment (*n* = 5). (L,N) HE staining and IHC images for CK‐19, and Ki67 in liver tissues from DEN/CCL_4_ and AKT/NRAS models following NAT10‐2023 treatment. (M,O) Quantification of IHC results for CK‐19 and Ki67 in and serum levels of AST and ALT in DEN/CCL_4_ and AKT/NRAS following NAT10‐2023 treatment. mice. Data in (C), (H), (J), (K), (M), and (O) are presented as the mean ± SD. ^*^ *p* < 0.05, ^**^ *p* < 0.01, ^***^ *p* < 0.001, ^****^ *p* < 0.0001, ns., not significant. Two‐tailed Student's *t*‐test.

To investigate the therapeutic potential of NAT10‐2023 in HCC, we conducted pharmacodynamic evaluations both in vitro and in vivo. In vitro assays using CCK8 were performed on human liver stellate cells (LX2), human embryonic kidney cells (HEK293), and liver cancer cells (HCCLM3 and SK‐HEP‐1). The half maximal inhibitory concentration (IC50) values revealed a selective effect of NAT10‐2023 on cancer cell viability, with significantly lower IC50 values for SK‐HEP‐1 and HCCLM3 compared to LX2 and HEK293, suggesting a strong tumor‐targeting capability (Figure 8A). Following this observation, we examined the effect of NAT10‐2023 on SMAD3 expression levels at varying concentrations. WB analysis revealed a dose‐dependent downregulation of SMAD3 expression in response to NAT10‐2023 treatment (Figure 8B). Based on these findings, we selected a concentration of 5 µM for subsequent experiments to investigate its effects on key cellular behaviors, specifically focusing on colony formation, migration, and invasion. Colony formation and transwell assays revealed that NAT10‐2023 significantly suppressed HCC cell proliferation, invasion, and migration at this concentration (Figure 8C and Figure S8A,B). Moreover, apoptosis assays demonstrated a dose‐dependent increase in both apoptotic and anoikis rates in HCCLM3 and SK‐HEP‐1 cells following NAT10‐2023 treatment, with a significant effect observed at higher concentrations (10 µM). Notably, NAT10‐2023 treatment resulted in a substantially higher rate of anoikis compared to apoptosis, further highlighting its efficacy in disrupting tumor cell adhesion. (Figure S8C–F). These results above support the efficacy of NAT10‐2023 in inhibiting the tumor characteristics of HCC cells in vitro.

For in vivo validation of these findings, we established a mouse xenograft model to evaluate the antitumor effects of NAT10‐2023. Mice received NAT10‐2023 (10 mg kg^−1^) intervention via intraperitoneal injection once weekly, starting 12 days after subcutaneous tumor inoculation. This treatment significantly inhibited tumor growth, resulting in a marked reduction in tumor weight compared to controls (Figure 8D–F). Furthermore, bioluminescence imaging and analysis of the intraperitoneal metastasis model demonstrated reduced tumor metastasis and growth in the NAT10‐2023‐treated group, highlighting its efficacy in suppressing tumor activity (Figure 8G,H). In addition to the xenograft model, we evaluated the effect of NAT10‐2023 on hepatocarcinogenesis in the DEN/CCL_4_ chemically induced model and the AKT/NRAS oncogene‐driven model (Figure 8I). In both settings, NAT10‐2023 treatment markedly suppressed HCC progression, as evidenced by fewer and smaller liver tumors compared to controls (Figure 8J,K). Histological examination via HE staining revealed a decreased tumor burden, while IHC analysis showed significantly lower expression levels of tumor biomarkers CK‐19 and Ki67 (Figure 8L–O). WB results further confirmed that NAT10‐2023 treatment significantly reduced CK‐19 expression, supporting its antitumor effects (Figure S8G,H). Moreover, serum analyses demonstrated improved liver function in NAT10‐2023‐treated mice, with significantly lower AST and ALT levels compared to controls (Figure 8M,O). Sirius Red staining in the DEN/CCL_4_‐induced model further revealed that NAT10‐2023 significantly alleviated liver fibrosis progression (Figure S8I). This antifibrotic effect was accompanied by significant reductions in serum AFP levels and pro‐inflammatory cytokines, including IL‐1β and IL‐6, as determined by ELISA analysis (Figure S8J,K). These findings suggest that NAT10‐2023 not only reduces tumor biomarkers but also alleviates fibrosis by attenuating chronic inflammation. Importantly, NAT10‐2023 treatment had no significant impact on mouse body weight in both the DEN/CCL_4_ and AKT/NRAS models, further supporting its safety (Figure S8L,M). In conclusion, these results collectively demonstrate that NAT10‐2023 exhibits strong therapeutic potential for liver cancer, effectively inhibiting tumor growth, reducing metastasis, suppressing hepatocarcinogenesis, preventing fibrosis, and preserving liver function across various preclinical models.

## 3 Discussion

**FIGURE 9:**
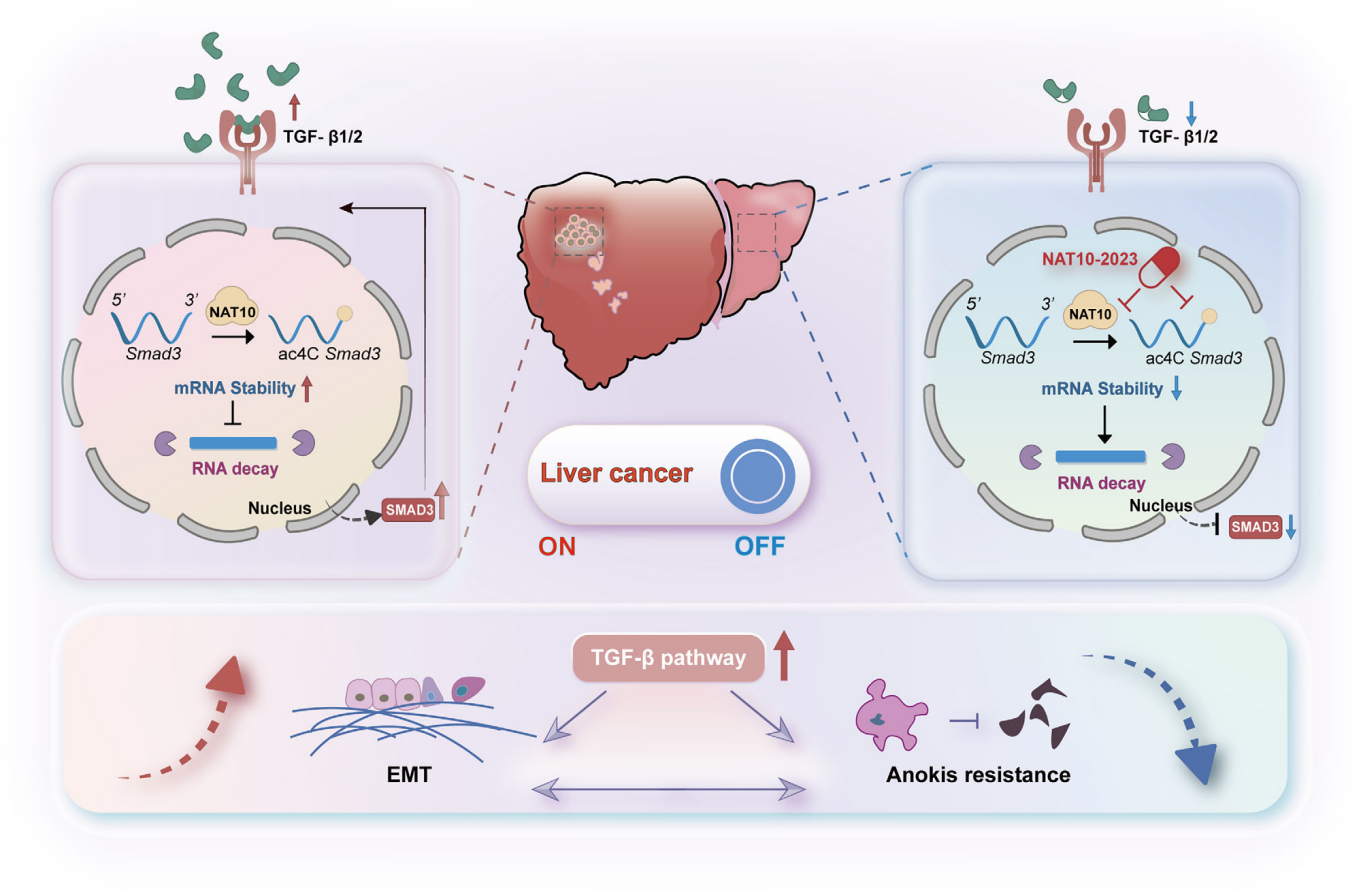
Model of NAT10 function in HCC progression. The model illustrates the functional mechanisms of NAT10 in HCC progression through mRNA ac4C modification of SMAD3. Elevated NAT10 expression stabilizes SMAD3 mRNA via ac4C modification, facilitating tumor progression. Additionally, NAT10 facilitates the activation of the TGF‐β signaling pathway, which contributes to the EMT and inhibits anoikis. Furthermore, the small‐molecule inhibitor NAT10‐2023 disrupts NAT10's interaction with RNA, thereby reducing RNA ac4C modification levels and suppressing HCC progression.

In this study, we elucidated the critical role of NAT10 in the development and progression of HCC, revealing its mechanisms of action through epitranscriptomic regulation (Figure 9). NAT10 has been implicated in various oncogenic processes through RNA acetylation; however, its specific role in HCC progression remains unclear. Our findings highlight two significant innovations. First, we demonstrate that NAT10 enhances the stability of SMAD3 mRNA through ac4C modification, which is crucial for promoting HCC progression. Elevated NAT10 expression stabilizes SMAD3 mRNA via this modification, facilitating tumor progression and contributing to the activation of the TGF‐β signaling pathway. This pathway plays a pivotal role in EMT and inhibits anoikis, further driving tumor malignancy. Using advanced techniques, including single‐base resolution RNA ac4C detection via Reda C:T‐seq and NAT10 RIP‐seq, we confirmed the involvement of SMAD3 as a key downstream target of NAT10. These findings shed light on the intricate relationship between RNA modifications and tumorigenesis, emphasizing the role of NAT10 in regulating oncogenic signaling pathways. This is consistent with previous studies showing NAT10‐mediated ac4C modification of oncogenic transcripts. For example, NAT10 stabilizes heat shock protein 90 alpha family class A member 1 (HSP90AA1) mRNA via ac4C modification in HCC [14]. Furthermore, NAT10 enhances mutant p53 levels by antagonizing MDM2 activity independently of RNA acetylation [34]. Together, these results support a broader role of NAT10 in stabilizing oncogenic transcripts in HCC, with SMAD3 identified as a key target in this study.

Second, we identified a novel small‐molecule inhibitor, NAT10‐2023, which effectively blocks NAT10 activity. Our data indicate that NAT10‐2023 not only inhibits NAT10 function but also exhibits potent antitumor characteristics both in vitro and in vivo. Importantly, NAT10‐2023 disrupts NAT10's interaction with RNA, thereby reducing RNA ac4C modification levels and suppressing HCC progression. This small molecule may serve as a valuable tool for further research into the role of NAT10 in liver cancer and potentially in other NAT10‐related diseases, marking a promising therapeutic strategy for HCC treatment.

Moreover, our research highlights the previously unreported role of NAT10‐mediated RNA ac4C modification in both the occurrence of HCC and the regulation of anoikis resistance. While prior studies have suggested that NAT10 may promote HCC progression and drug resistance through RNA ac4C modification or independent pathways [22, 29], these studies did not adequately address NAT10's critical role in the dynamic process of liver cancer development. Our findings demonstrate that knockdown of NAT10 or inhibition of its activity using small molecules NAT10‐2023 effectively suppresses tumor formation in spontaneous liver cancer models while mitigating liver damage associated with tumor progression. Additionally, our findings underscore the regulatory role of NAT10 in anoikis, a form of programmed cell death triggered when adherent cells lose attachment to the extracellular matrix. Anoikis is crucial for processes such as embryonic development, tumor progression, metastasis, and drug resistance [35, 36]. While previous studies have linked NAT10 to apoptosis, ferroptosis, and cellular senescence, its precise involvement in anoikis regulation has remained unexplored. Our results suggest that NAT10 regulates anoikis resistance in HCC cells by modulating key genes, including SMAD3 [37, 38], thus facilitating tumor cell EMT and enhancing malignant progression.

Given that NAT10 is a potential therapeutic target for HCC treatment, further exploration of inhibitors is of great significance for clinical applications. In our study, we conducted rigorous virtual screening approaches and functional validation to demonstrate that the small molecule NAT10‐2023 could effectively suppress the progression of HCC both in vitro and in vivo. Compared to the previously reported small molecule Remodelin, NAT10‐2023 shows greater efficacy in inhibiting intracellular RNA ac4C modification and NAT10‐RNA interactions at lower concentrations. Using the Schordinger software, we further compared NAT10‐2023 with two previously reported NAT10 inhibitors, paliperidone and AG‐401, to assess their binding affinities to NAT10 [20]. Our results show that NAT10‐2023 exhibits significantly lower binding energy to NAT10, indicating its superior binding efficiency. In subsequent experiments, we selected a concentration of 5 µM to evaluate the effects of NAT10‐2023 on SMAD3 expression levels and other key cellular behaviors. In vivo, we tested various doses of NAT10‐2023 (1, 5, 10, 15, and 20 mg kg^−1^, administered weekly via intraperitoneal injection). A dose‐dependent antitumor effect was observed at 1, 5, and 10 mg kg^−1^, with the optimal results at 10 mg kg^−1^. However, higher doses (15 and 20 mg kg^−1^) led to mouse mortality, which led to the selection of 10 mg kg^−1^ for further studies. At this dose, significant inhibition of tumor growth, metastasis, and hepatocarcinogenesis was observed. These results highlight the potential of NAT10‐2023 as a promising therapeutic agent for targeting the NAT10‐SMAD3 regulatory axis in HCC, paving the way for new therapeutic strategies in the management of this aggressive cancer.

Despite these advancements, our study has several limitations. We did not fully explore the additional biological functions of NAT10 that may be crucial in HCC, as our focus was primarily on RNA ac4C modification. NAT10 is also involved in other molecular processes, such as histone acetylation and microtubule regulation [39, 40]. Future studies should aim to delineate the specific pathways through which NAT10 exerts its effects on HCC. Additionally, while we confirmed the interaction of NAT10‐2023 with NAT10 using various methodologies, structural data such as crystallography or cryo‐electron microscopy would further enhance our understanding of its binding dynamics.

In conclusion, our findings not only elucidate the oncogenic role of NAT10 in HCC through ac4C‐mediated regulation of SMAD3 mRNA stability but also provide a promising avenue for therapeutic intervention with NAT10‐2023. These insights significantly contribute to the understanding of liver cancer mechanisms and highlight the potential for novel therapeutic strategies targeting NAT10‐related pathways.

## 4 Conclusion

Our findings demonstrate that NAT10 drives HCC progression by stabilizing SMAD3 mRNA via ac4C modification, thereby activating the TGF‐β signaling pathway. NAT10‐2023 represents a promising small‐molecule therapeutic candidate for targeting NAT10 in HCC treatment.

## 5 Materials and Methods

### 5.1 Study Design

This study aimed to elucidate the critical mechanisms by which the RNA acetyltransferase NAT10 is involved in the initiation of HCC, anoikis resistance, and the metastatic progression of liver cancer. Additionally, we sought to screen for potential small‐molecule inhibitors of NAT10 and validate their inhibitory effects on HCC progression both in vitro and in vivo. All in vitro experiments (except RIP‐seq and Reda C:T‐seq) were performed in triplicate. The number of animals used in in vivo experiments is indicated in the figure legends. In drug intervention experiments, mice were subjected to a double‐blind design prior to treatment to ensure uniform tumor burden distribution across groups. The pathological testing experiments and analyses were conducted by a third‐party testing agency, and the experimental personnel were blind to the group assignments.

### 5.2 Plasmid Construction and Lentiviral Infection

shCtrl (shRNA targeting sequence: CAACAAGATGAAGAGCACCAA) and shNAT10 (shRNA targeting sequence: GCAATTGTACACAGTGACTAT) sequences were integrated into the pLKO.1‐hygro plasmid. The NAT10 synonymous mutant (sequence: ACAGCTGTACACCGTGACTAT) was integrated into the pcDNA3.0 plasmid. HEK293T cells were seeded into 10 cm dishes and transfected using Lipofectamine 2000 with pMD2.G, psPAX2, and either pLKO.1 or pLenti‐CMV plasmids containing the shRNA constructs. Lentivirus was collected at 48 h post‐transfection. Cells were then infected with the collected lentivirus for 48 h and selected with antibiotics for 3 days to establish stable cell lines.

### 5.3 Clinical Sample Sources

This study utilized liver cancer TMA samples (HLivH180Su11) from Shanghai Biochip Company, including 95 liver cancer specimens, of which 87 had corresponding adjacent non‐cancerous tissue samples. Additionally, 28 surgical resection specimens from patients with liver cancer at Taihe Hospital in Shiyan were included. The research involving human samples was approved by the Ethics Committee of Hubei University of Medicine, and informed consent was obtained from all patients.

### 5.4 Cell and Animal Models

Cell lines used in this project were obtained from Beijing Biocytogen and Saiweier Biotechnology, all of which underwent STR authentication to ensure correct cell origin and PCR testing to confirm the absence of mycoplasma contamination.

The DEN/CCL_4_‐induced HCC model was established using male C57BL/6 mice [41]. At 3 weeks of age, mice received a single intraperitoneal injection of 25 mg kg^−1^ DEN. From weeks 4 to 23, mice were administered CCL_4_ (5 µL g^−1^, 10% CCL_4_) biweekly. In studies validating the impact of NAT10 expression silencing on liver cancer development, adenoviruses were administered via the tail vein at weeks 8 and 20, with euthanasia at week 36. In drug studies, NAT10‐2023 was administered via intraperitoneal injection (10 mg kg^−1^) once weekly from weeks 8 to 32.

For the AKT/NRAS‐induced HCC model [42], male C57BL/6 mice received a mixture of plasmids (pT3EF1a‐AKT‐HA, 2 µg/pCAG‐NRAS, 8 µg/SB100, 1 µg) via the tail vein at week 6. To validate the effects of NAT10 silencing on liver cancer suppression, shNAT10‐AAV was administered via the tail vein at week 9, followed by euthanasia at week 15. To assess the antitumor activity of the NAT10 small‐molecule inhibitor, NAT10‐2023 was administered (10 mg kg^−1^) via intraperitoneal injection from weeks 10 to 14. Following euthanasia, liver tissues were harvested for imaging and analysis using ImageJ software for tumor area and number counting. Serum samples were collected for AST and ALT detection (using the Solexa method) and for histopathological examination.

Immunodeficient mouse models were constructed for subcutaneous tumor models using BALB/c‐nu mice. For the subcutaneous tumor model, HCC cells were resuspended in serum‐free medium at 1 × 10^7^ cells/100 µL and injected subcutaneously into the mice. For the drug intervention model, small molecules were administered intraperitoneally (10 mg kg^−1^) starting 1 week after cell inoculation. For the intraperitoneal metastatic models, to objectively assess the impact of NAT10 silencing on HCC metastatic potential, highly immunodeficient mice were utilized. HCC cells were resuspended in serum‐free medium at 2 × 10^6^ cells/200 µL and injected intraperitoneally. Observations were conducted using IVIS imaging, and mouse survival was monitored.

### 5.5 NAT10 RIP and acRIP Analyses

For RIP‐seq experiments, 2 × 10^7^ HCC cells were lysed in 1 mL IP buffer (1 × PBS + 0.5% NP‐40, with 5 µL RNAase inhibitor and 5 µL protease inhibitor). The lysate was subjected to sonication (five times at 10% power) and centrifuged at 13,000 rpm for 10 min at 4°C. The supernatant was incubated with 3 µg of NAT10 monoclonal antibody at 4°C for 5 h, followed by the addition of 20 µL of pre‐blocked protein A/G beads for an additional 2 h. The complex was washed five times with IP buffer, and RNA was extracted using Trizol for next‐generation sequencing analysis or RT‐qPCR.

For acRIP experiments, 2 mg of RNA was extracted using Trizol, resuspended in RNA‐IP buffer (50 mM Tris pH 7.4, 100 mM NaCl, 0.05% NP40), and incubated with 3 µg of ac4C monoclonal antibody at 4°C for 5 h. Protein A/G beads were added for another 2 h, followed by five washes with IP buffer to remove non‐specific binding. RNA was then extracted using Trizol for RT‐qPCR analysis.

### 5.6 Reda C:T‐seq and RNA‐seq

Following previously reported methods for Reda C:T‐seq [43], 10 µg of RNA was treated to remove rRNA and subsequently subjected to 2 mM sodium borohydride treatment (at 55°C for 1 h) before constructing cDNA libraries and sequencing.

RNA‐seq library preparation and analysis were conducted following previously established protocols [44] and were performed by Novogene Company.

### 5.7 Luciferase Assay

SMAD3 3′UTR reporter constructs, including wild‐type (SMAD3‐WT) and mutant (SMAD3‐MT) versions (where all five ac4C sites were mutated), were synthesized and cloned into the psiCHECK 2.0 vector. The luciferase plasmids were co‐transfected into HEK293T cells with siRNA using Lipofectamine 2000. Forty‐eight hours post‐transfection, firefly luciferase activity was measured using the Double‐Luciferase Reporter Assay Kit (Promega, USA). Renilla luciferase activity was used for normalization, and bioluminescence was detected with an MD SpectraMax i3x system.

### 5.8 mRNA Stability Assay

HCCLM3 and SK‐HEP‐1 cells were treated with 10 µg mL^−1^ Actinomycin D to inhibit transcription. Cells were collected at the following time points: 0, 1, 2, 4, 6, and 8 h after treatment. RNA was extracted from the treated cells using TRIzol reagent following the manufacturer's instructions. cDNA was synthesized using a standard reverse transcription protocol. SMAD3 mRNA expression levels were quantified by qPCR, with ACTB serving as the internal control for normalization. The relative abundance of SMAD3 mRNA at each time point was calculated, and mRNA decay curves were constructed. The decay rate and half‐life of SMAD3 mRNA were determined through non‐linear regression curve fitting using GraphPad Prism software.

### 5.9 Protein Expression Purification, MST, and CETSA Experiments

Using a synthetic gene assembly method, we constructed the NAT10 expression sequence, which was then cloned into the P35 expression vector. Plasmids were amplified in DH5α bacteria and subsequently transfected into HEK293 cells. After 5 days, cells were lysed in a buffer (50 mM Tris, 150 mM NaCl [pH 8.0], 8 M urea, 20 mM imidazole), and NAT10 was purified using Ni‐IDA affinity chromatography. After purification, the protein was refolded in a dialysis bag under specified conditions for 6–8 h. The purified protein was subjected to freeze‐thaw experiments, SDS‐PAGE, and WB to confirm its purity and quality.

For CETSA experiments, NAT10‐2023 (5 µM) or an equal volume of DMSO was co‐incubated with HCC cells for 4 h. Cells were collected in pre‐chilled PBS and subjected to a temperature gradient (36.3–55°C) for 3 min using a Bio‐Rad T100 thermal cycler. Soluble proteins were extracted using liquid nitrogen freeze‐thaw methods, followed by western blot analysis for NAT10 and ACTB expression.

For MST experiments, recombinant NAT10 was labeled using the NanoTemper RED‐NHS protein labeling kit (MO‐L011). The labeled protein was mixed with varying concentrations of small‐molecule compounds, and interactions were measured according to the instrument's operational protocols.

### 5.10 Virtual Screening and Molecular Dynamics Simulations

We obtained the human NAT10 spatial structure from AlphaFold2 [45]. Based on the MCE_Anti‐cancer Compound Library_2021‐05‐28 and AutoDock 4.2.6 software [46], we performed virtual screening to predict the binding interactions of small molecules with NAT10 and identified key residues such as ILE629, GLY639, GLY641, LEU719, and PHE722 for binding potential.

For molecular dynamics simulations, we prepared the NAT10 receptor using PDBFixer and AmberTools 20's tLEaP module to repair missing atomic residues [47]. The AM1‐BCC charge for the compound NAT10‐2023 was calculated using the Amber antechamber program. Subsequently, we conducted molecular dynamics simulations using the AMBER ff19SB and GAFF force fields.

### 5.11 Statistical Analysis

Statistical analysis was performed using Student's two‐tailed *t*‐test or one‐way ANOVA with Tukey's test. Statistical images were plotted using GraphPad Prism 8 statistical software. Survival curves and their *p*‐values were determined using the Kaplan–Meier method and the log‐rank test, respectively. Univariate and multivariate Cox regression analyses were conducted to assess the significance of various variables for survival using SPSS 19.0 statistical software. All data are presented as means ± SD. The *p*‐values were determined as *p < 0.05, **p < 0.01, ***p < 0.001, ****p < 0.0001, and n.s. (not significant).

## Conflicts of Interest

The authors declare no conflicts of interest.

## Supporting information




**Supporting Information file 1**: exp270074‐sup‐0001‐SuppMat.docx
