# Supplementary material for: Targeting NAT10 Inhibits Hepatocarcinogenesis via ac4C‐Mediated SMAD3 mRNA Stability
Source: Exploration (Beijing). 2025 Sep 4;5(6):20250075. doi: 10.1002/EXP.20250075 (PMC12752639; doi:10.1002/EXP.20250075)
Supplement: Supplementary file 1 — Supporting Information file 1: exp270074‐sup‐0001‐SuppMat.docx [file EXP2-5-20250075-s001.docx]

Targeting NAT10 inhibits hepatocarcinogenesis via ac4C-mediated SMAD3 mRNA stability

**Supplementary Materials**

**
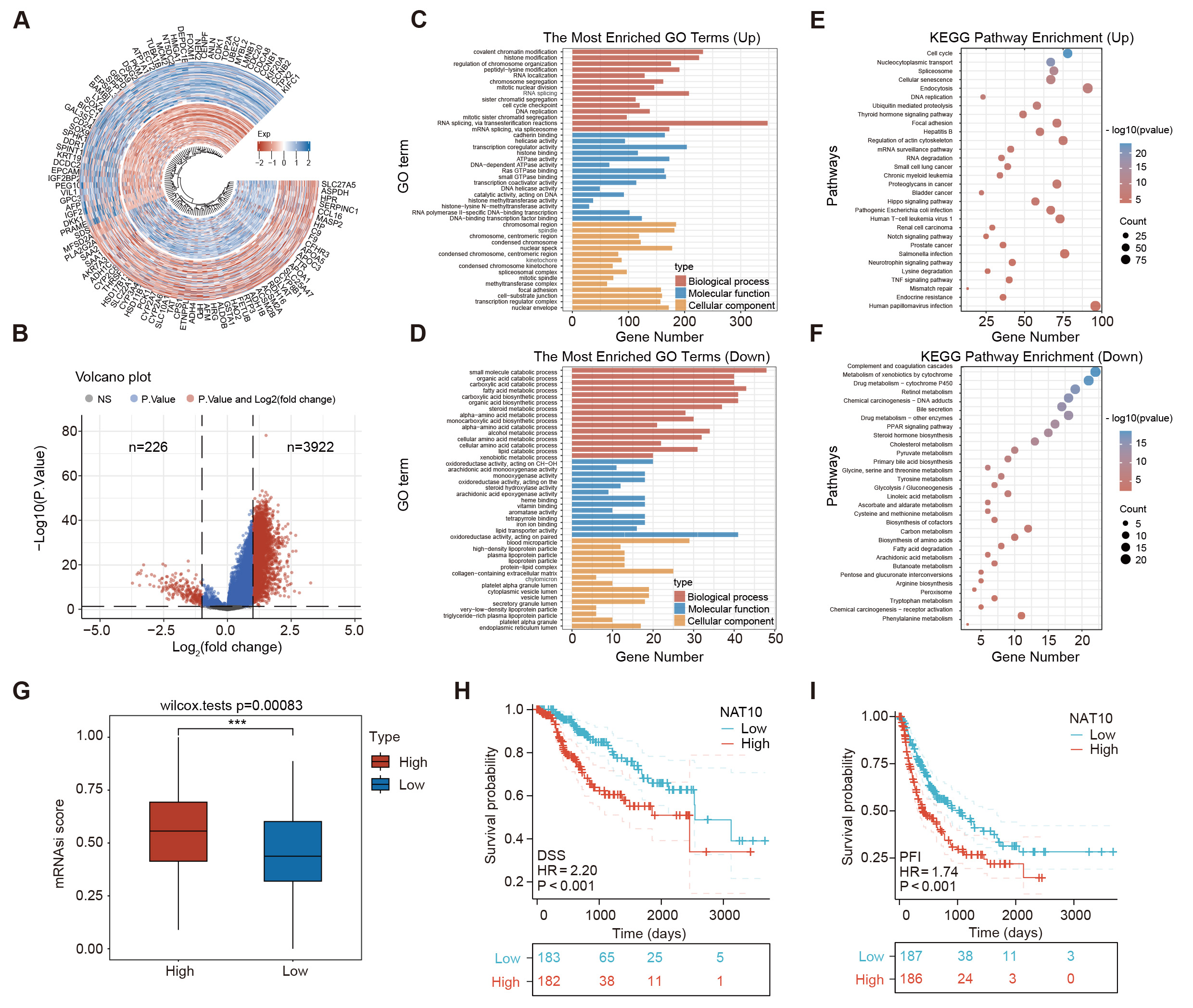
**

**Figure S1**

Transcriptomic analysis of NAT10 in HCC and association of NAT10 expression with mRNAsi and patient outcomes. (A) Heatmap displaying the expression profiles of genes associated with NAT10 in HCC from TCGA dataset, where red indicates upregulation and blue indicates downregulation. (B) Volcano plot illustrating differential gene expression of NAT10 related genes, highlighting significant upregulated (n = 3922) and downregulated (n = 226) genes based on log2 fold change and p-value. (C-D) Bar chart of enriched Gene Ontology (GO) terms for upregulated and downregulated genes. (E-F) Dot plot showing KEGG pathway enrichment for upregulated and downregulated genes. (G) Boxplot showing mRNA stemness index (mRNAsi) scores between high NAT10 expressionand low NAT10 expression. (H-I) Kaplan-Meier survival curves indicating disease-specific survival (DSS) and progress-free intervals (PFI) based on NAT10 expression levels.


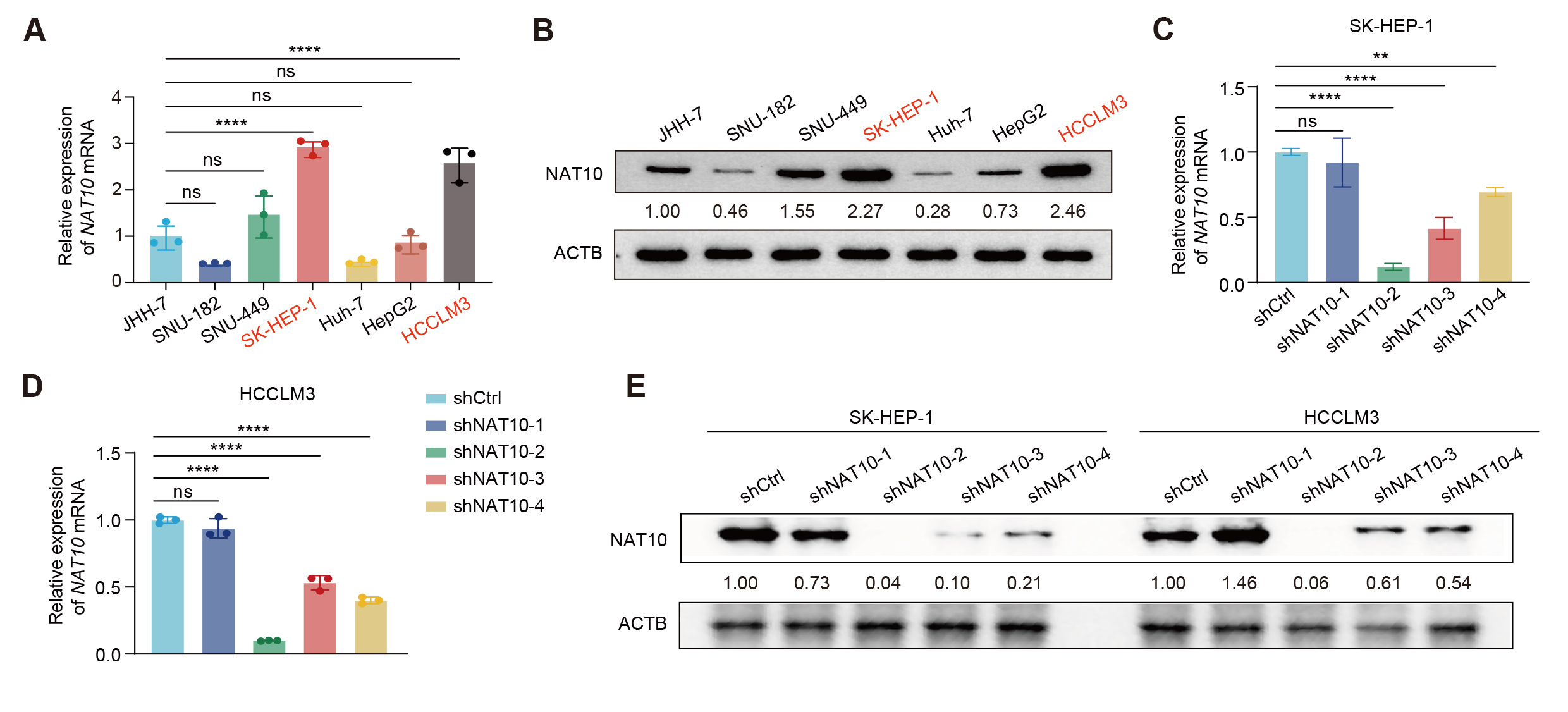


**Figure S2**

Expression levels of NAT10 in HCC cell lines and confirmation of knockdown efficiency. (A) qPCR analysis showing relative NAT10 mRNA expression levels in different HCC cell lines. (B) WB analysis confirming NAT10 protein expression levels in different HCC cell line. (C-D) qPCR results demonstrating the efficiency of shRNA-mediated NAT10 knockdown in SK-HEP-1 and HCCLM3 cells. (E) WB analysis confirming the knockdown of NAT10 expression in SK-HEP-1 and HCCLM3 cells. Data in A and C-D are presented as the mean ± SD (n = 3). ^**^p < 0.01, ^****^ p < 0.0001, ns., not significant. One-way ANOVA with Tukey's test.


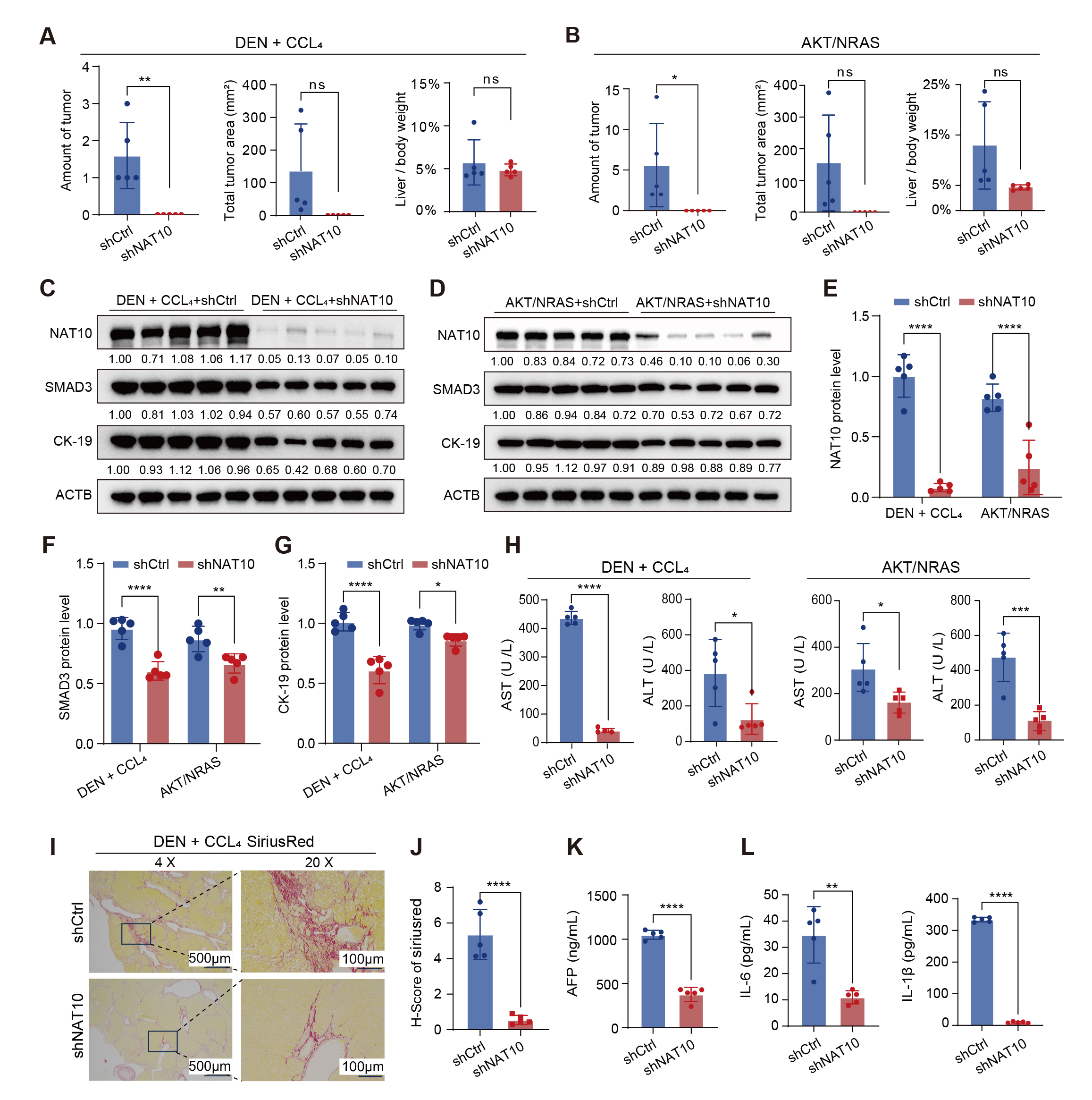


**Figure S3**

Evaluation of NAT10 expression and the effect of NAT10 on liver fibrosis in animal models. (A-B) Quantification of the amount of tumor (left), the total tumor area (middle) and the ratio of liver weight to body weight (right) in the DEN/CCL_4_ and AKT/nRAS models upon NAT10 knockdown (n = 5). (C-G) WB analysis showing NAT10, SMAD3 and CK-19 expression levels in liver tissue samples from DEN/CCL_4_ and AKT/nRAS-induced hepatocarcinogenesis animal models following NAT10 knockdown. (H) Quantification of serum levels of AST and ALT in DEN/CCL_4_ and AKT/NRAS and AKT/NRAS models following NAT10 knockdown. (I-J) Sirius Red staining evaluating liver fibrosis in the DEN/CCL_4_-induced model following NAT10 knockdown (K-L) ELISA analysis showing serum AFP levels (K) and pro-inflammatory cytokines IL-6 and IL-1β (L) in the DEN/CCL_4_-induced model following NAT10 knockdown. Data in A-B, E-H and J-L are presented as the mean ± SD. p < 0.05, ^**^p < 0.01, ^***^ p < 0.001, ^****^ p < 0.0001, ns., not significant. Two-tailed student’s t-test.


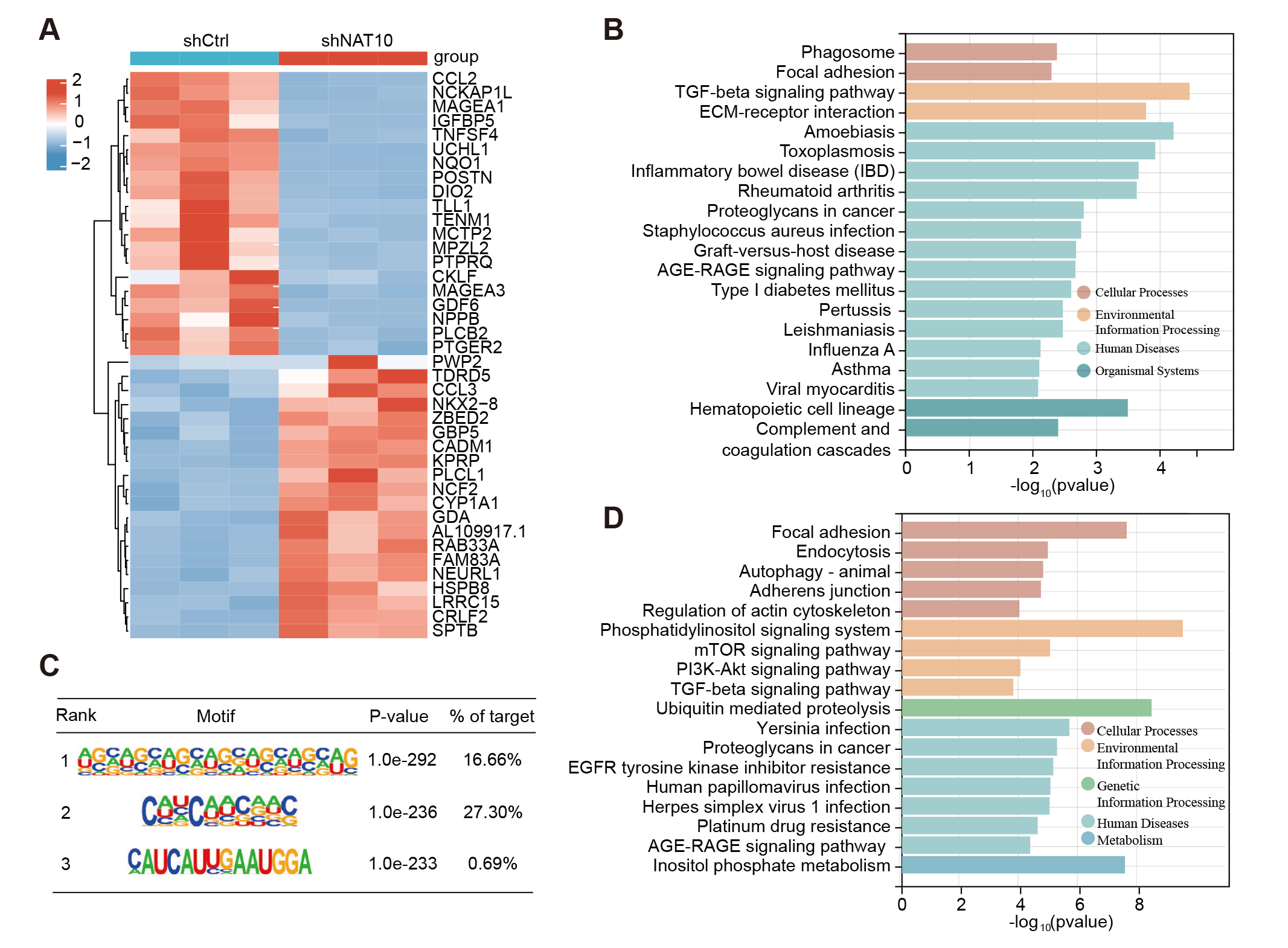


**Figure S4**

Analysis of NAT10 knockdown RNA-seq and NAT10 RIP-seq in SK-HEP-1 cells. (A) Heatmap illustrating the top 20 DEGs in NAT10-depleted SK-HEP-1 cells. (B) KEGG pathway enrichment analysis of downregulated DEGs in NAT10-depleted SK-HEP-1 cells. (C) Motif analysis showing enrichment of “CXXC” motif in NAT10 binding sequences by HOMER software. (D) KEGG pathway enrichment analysis of NAT10-targeted mRNAs in SK-HEP-1 cells.


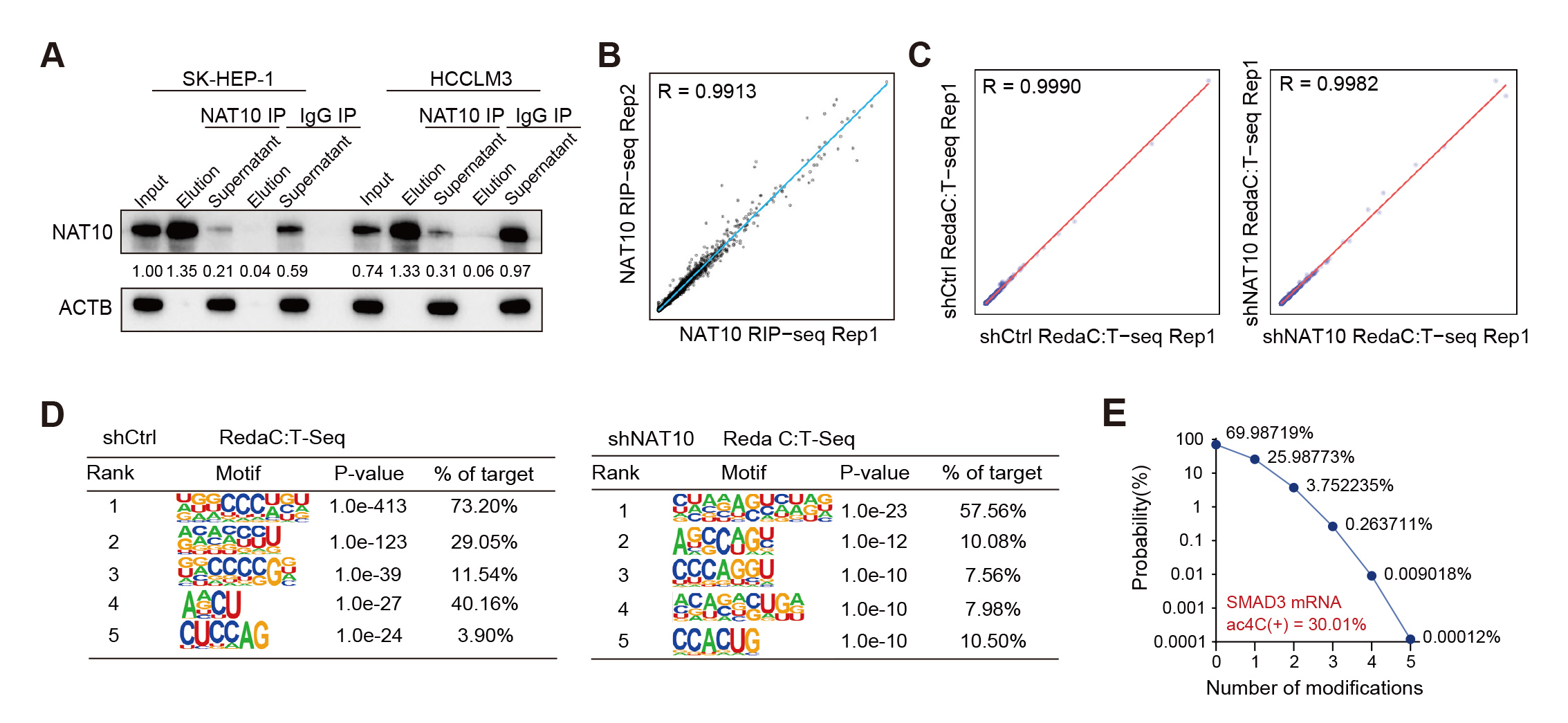


**Figure S5**

Evaluation of NAT10 IP efficiency, library reproducibility, and abundance of SMAD3 mRNA ac4C modifications. (A) IP efficiency of NAT10 antibody in SK-HEP-1 and HCCLM3 cells. (B) Correlation of NAT10 RIP-seq biological replicates in SK-HEP-1 cells. (C) Correlation of Reda C:T-seq biological replicates in shCtrl and shNAT10 SK-HEP-1 cells. (D) Sequence motifs enriched at ac4C modification sites in control and NAT10-depleted cells by HOMER software. (E) SMAD3 mRNA ac4C modification abundance.


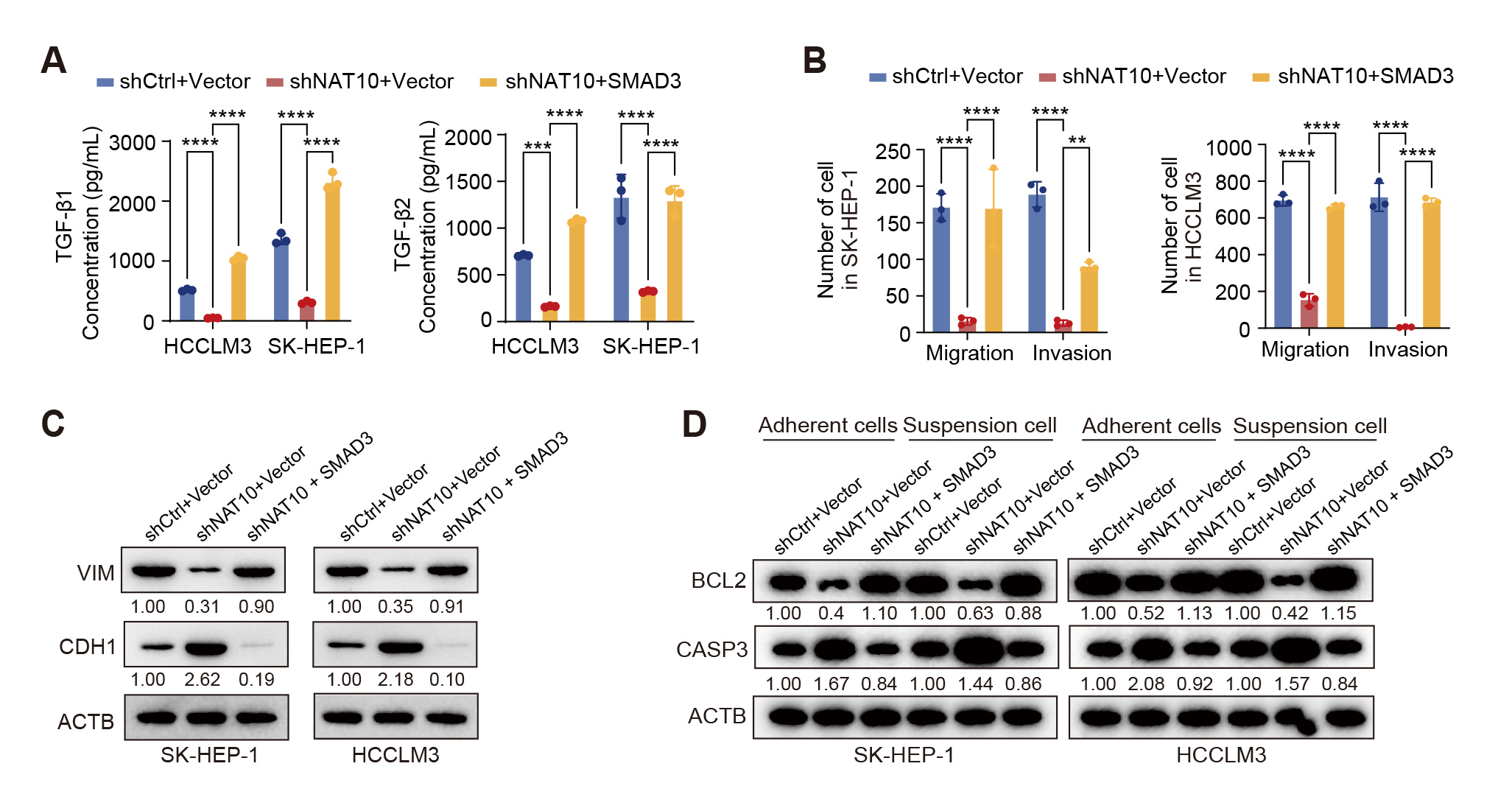


**Figure S6**

The effect of NAT10-SMAD3 regulatory axis on HCC cell progression. (A) ELISA analysis showing the expression levels of TGF-β1 and TGF-β2 after NAT10 knockdown and SMAD3 rescue in SK-HEP-1 and HCCLM3 cells. (B) Quantification of migration and invasion assays in SK-HEP-1 and HCCLM3 cells with NAT10 knockdown and SMAD3 rescue. (C) WB analysis showing the expression levels of CDH1 and VIM protein in SK-HEP-1 and HCCLM3 cells with NAT10 knockdown and SMAD3 rescue. (D) WB analysis showing the expression levels of BCL2 and CASP3 in SK-HEP-1 and HCCLM3 cells with NAT10 knockdown and SMAD3 rescue. Data in A and B are presented as the mean ± SD. ^**^p < 0.01, ^***^p < 0.001, ^****^ p < 0.0001. One-way ANOVA with Tukey's test.


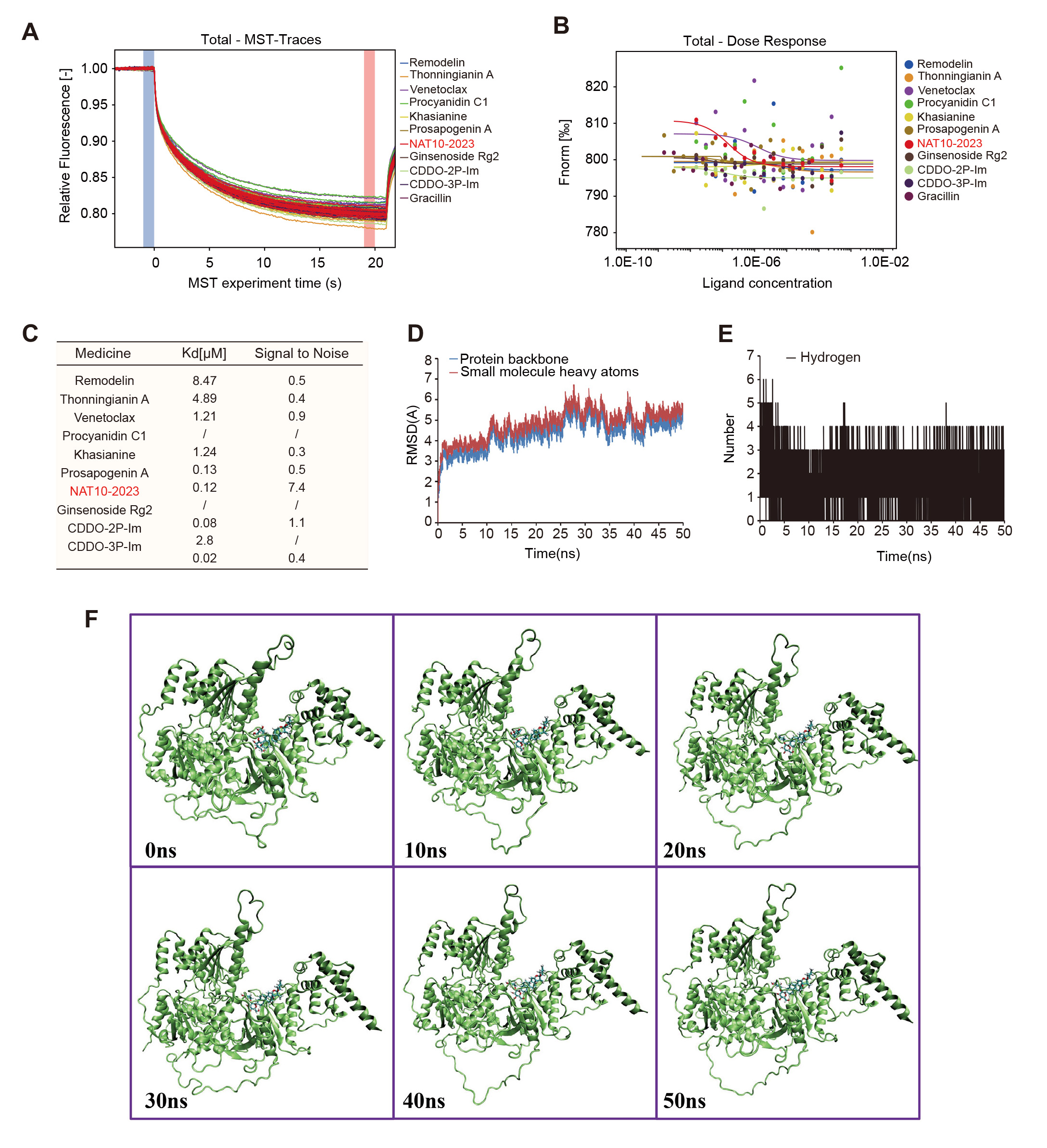


**Figure S7**

Identification NAT10-2023 as a novel NAT10 inhibitor. (A) MST traces for tested compounds, indicating binding interactions with NAT10. (B) Dose-response curves from MST assays showing the binding affinity of tested compounds to NAT10. (C) Table summarizing the Kd and signal-to-noise ratios for key compounds. (D) RMSD analysis from molecular dynamics simulations of the NAT10-NAT10-2023 complex over time. (E) Hydrogen bond frequency analysis over the simulation trajectory. (F) Snapshots from molecular dynamics simulations showing conformational changes in NAT10 upon NAT10-2023 binding.


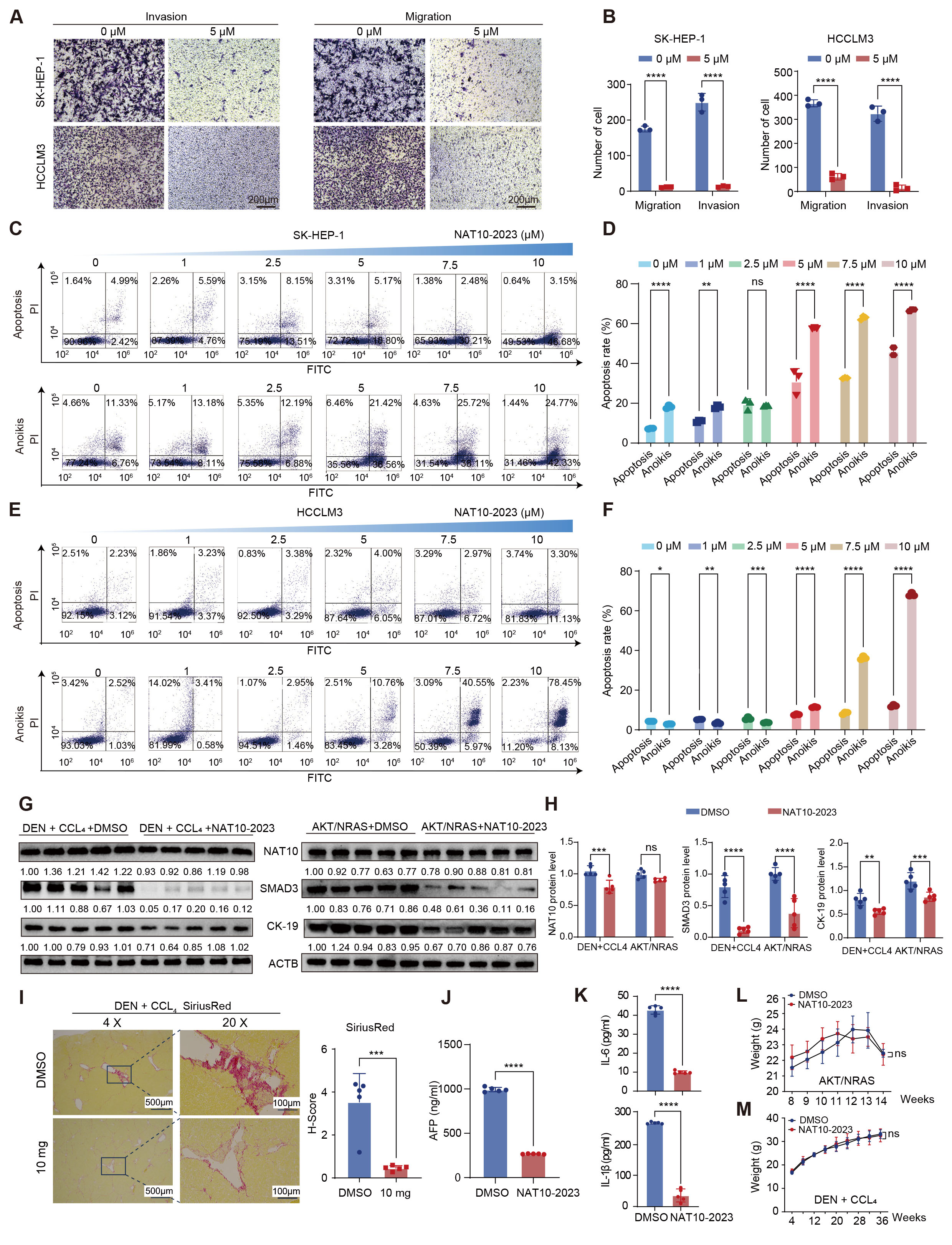


**Figure S8**

NAT10-2023 inhibits HCC cell invasion, migration, and induces apoptosis and anoikis. (A-B) Transwell invasion and migration assays of HCCLM3 and SK-HEP-1 cells treated with 5 µM NAT10-2023. (C-F) Flow cytometric analysis of apoptosis and anoikis in HCCLM3 and SK-HEP-1 cells treated with increasing concentrations of NAT10-2023. (G-H) WB analysis showing NAT10, SMAD3 and CK-19 expression levels in liver tissue samples from DEN/CCL_4_ and AKT/nRAS-induced animal models following NAT10-2023 treatment. (I) Sirius Red staining of liver fibrosis in DEN/CCL_4_ mice treated with NAT10-2023. (J-K) ELISA analysis showing serum AFP levels and pro-inflammatory cytokines IL-6 and IL-1β in the DEN/CCL_4_-induced model mice treated with NAT10-2023. (L-M) Body weight monitoring of AKT/nRAS and DEN/CCL_4_ model mice treated with NAT10-2023. Data in B, D, F and H-M are presented as the mean ± SD. ^*^p < 0.05, ^**^p < 0.01, ^***^p < 0.001, ^****^ p < 0.0001, ns., not significant. Two-tailed student’s t-test and One-way ANOVA with Tukey's test.

**Table S1. Key Residue Hydrogen Bond Data**

Key Residue Hydrogen Bond Data（Fraction > 1%）

| **Name** | **Frames** | **Fraction** | **Avg. Distance** | **Avg. Angle** | **Lifetimes** |
| --- | --- | --- | --- | --- | --- |
| SER536@O-LIG919@O5_H38 | 41778 | 0.8356 | 2.7264 | 159.2825 | 4152 |
| SER422@OG-LIG919@O9_H48 | 24593 | 0.4919 | 2.8032 | 158.116 | 4715 |
| LIG919@O4-ILE629@N_H | 14194 | 0.2839 | 2.914 | 161.2856 | 9062 |
| ILE629@O-LIG919@O4_H37 | 2501 | 0.05 | 2.6803 | 157.559 | 315 |
| LIG919@O3-TRP723@NE1_HE1 | 2276 | 0.0455 | 2.8953 | 151.8352 | 1416 |
| SER422@OG-LIG919@O7_H46 | 1511 | 0.0302 | 2.8503 | 150.2532 | 893 |
| LIG919@O7-SER422@OG_HG | 1141 | 0.0228 | 2.8061 | 159.8829 | 332 |
| SER422@OG-LIG919@O8_H47 | 822 | 0.0164 | 2.8125 | 150.796 | 270 |
| LIG919@O8-TYR538@N_H | 577 | 0.0115 | 2.9307 | 151.7473 | 515 |

**Note:**

1. LIG919 refers to the compound NAT10-2023.

2. The "Name" represents the residue pairs forming hydrogen bonds; "Frames" indicates the number of frames in which the hydrogen bond exists in the trajectory; "Fraction" is the proportion (calculated as Frames divided by the total sampling frames); "Avg. Distance" and "Avg. Angle" represent the average bond length and bond angle, respectively.

3. "SER536@O-LIG919@O5_H38" means the oxygen atom on protein residue SER536 forms a hydrogen bond with the hydrogen atom H38 on compound O5, and similarly for other entries.
